# Ultrasound Contrast Agent Priming of Biopsy and Introducer Needles by Using a Small Syringe to Improve Needle Visibility in a Phantom Model

**DOI:** 10.1007/s00270-023-03500-3

**Published:** 2023-07-12

**Authors:** Per Thunswärd, Karin Österberg, Håkan Ahlström

**Affiliations:** 1 Department of Surgical Sciences - Section of Radiology, Uppsala University, Uppsala University Hospital, Entrance 70, 1st Floor, S-751 85 Uppsala, Sweden; 2 Department of Radiology, Västmanlands Hospital Västerås, Västerås, Sweden; 3 Antaros Medical AB, Mölndal, Sweden

**Keywords:** Needle visibility, Ultrasound, Contrast-enhanced ultrasound, Contrast-specific imaging-mode, Needle priming, Ultrasound contrast agent, Needle filling, Core needle biopsy, Core biopsy needle, Introducer needle

## Abstract

**Purpose:**

Biopsy under the guidance of contrast-enhanced ultrasound is sometimes useful. Needle visualization in contrast-specific imaging-mode is often poor; however, it may be improved by priming the needles with an ultrasound contrast agent. This study aimed to evaluate needle priming methods using the ultrasound contrast agent sulfur hexafluoride and a 1 mL syringe.

**Material and Methods:**

Two kinds of biopsy needles, side-notch and full core, and one kind of introducer needle were primed using non-primed needles as controls (*n* = 180). Recordings of punctures were performed in a water bath phantom to which the ultrasound contrast agent had also been added. Contrast-specific imaging-mode needle visibility was evaluated for the entire needles and the needle tips, respectively, quantitatively by calculating the contrast-to-noise ratio and qualitatively via grading by three radiologists.

**Results:**

The contrast-to-noise ratio following the ultrasound contrast agent priming was superior compared to the controls for the entire needles of all three types (*p* < 0.001) and for the needle tips of the core biopsy needles and introducer needles (*p* < 0.001). However, the ratio was equal to the controls for the needle tips of the side-notch biopsy needles (*p* = 0.19). Needle visibility following the ultrasound contrast agent priming was qualitatively superior compared to the controls for both the entire needles and the needle tips, and the difference was considered clinically relevant by the assessors (*p* < 0.001).

**Conclusion:**

The ultrasound contrast agent needle priming methods described increased the contrast-specific imaging-mode needle visibility in a phantom model. Nonetheless, the results also need to be confirmed in vivo.

**Supplementary Information:**

The online version contains supplementary material available at 10.1007/s00270-023-03500-3.

## Introduction

Performing biopsies under the guidance of contrast-enhanced ultrasound (CEUS) may be valuable to avoid targeting necrotic (non-diagnostic) tumor areas and to enable a biopsy of focal lesions only visible in the contrast-specific imaging-mode (CEUS-mode) [1–7]. Dual-screen imaging is recommended, with the CEUS-mode image on one side to visualize the lesion, accompanied by a B-mode image on the other side to track the needle because of the inferior CEUS-mode needle visibility [7–9]. To avoid disrupting the ultrasound contrast agent (USCA) microbubbles, the dual-screen B-mode image is of a low mechanical index (MI) and thus, of a lower image quality than in the conventional B-mode [8]. This may cause an inferior overall biopsy needle visibility, making the CEUS biopsy procedure more difficult. The ex vivo/in vitro CEUS-mode core biopsy needle visibility may be improved by needle priming with the USCA sulfur hexafluoride [10]. The priming method used, however, is limited to detachable side-notch biopsy needles and somewhat complicated by requiring the biopsy needle disassembly and a temporary sharps holder. Improved needle visibility following the needle priming has also been demonstrated in vivo, albeit only in a case report in which the needle priming procedure comprised coating of the outer cannula and the inner stylet of a semiautomatic biopsy instrument requiring 1–2 mL of USCA [11]. To be clinically useful, we identified the need for simpler and more universal priming methods requiring small amounts of USCA.

Needle priming methods using 0.2–0.6 mL of USCA were developed for the two most common types of non-detachable biopsy needle types, side-notch and full core, and for introducer needles. The aim was to evaluate the CEUS-mode needle visibility after applying those needle priming methods.

## Materials and Methods

### Experimental Setting

**Fig. 1:**
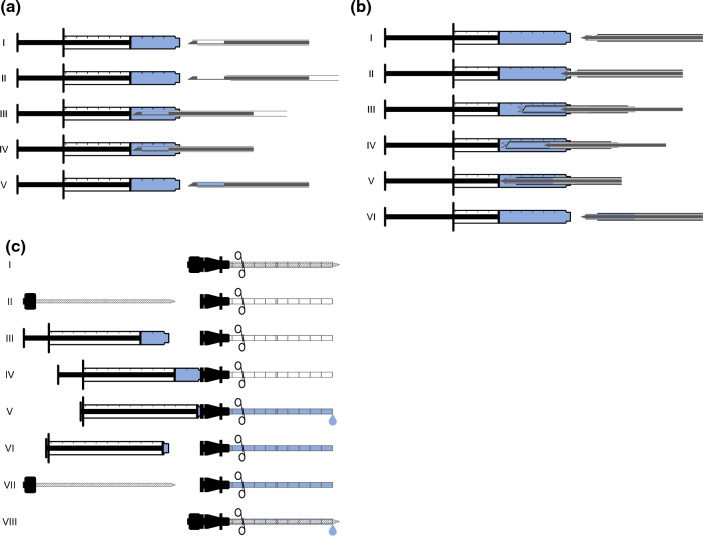
Schematic illustration of the three needle priming methods studied, all using a 1 mL syringe (the spaces between the inner stylets and the outer cannulas are intentionally magnified to illustrate the priming principle more clearly): **a** For the side-notch needle, 0.4 mL of the ultrasound contrast agent (USCA) was used, beginning with an unloaded instrument. In steps I–II, the first of the two charging steps was performed, exposing the distal part of the inner stylet, including the side-notch. In step III, the exposed distal part of the inner needle was inserted into the syringe. In step IV, the second of the two charging steps was performed, with the tip and syringe pointing upwards. In the final step, IV, the syringe was put back onto the table; furthermore, the instrument, including the needle, was withdrawn from the syringe and ready to fire off. **b** For the full core needle, 0.6 mL of the USCA was used, beginning with a loaded instrument with the stroke length set to 33 mm (not depicted). In steps I–II, the needle was inserted with the tip right inside the orifice of the syringe. In step III, the instrument was fired off into the syringe (separate automatic pincer step not depicted). In steps IV–V, the instrument was again loaded with the tip and the syringe pointing upwards. In the final step, VI, the syringe was put back onto the table; moreover, the instrument, including the needle, was withdrawn from the syringe and ready to fire off. **c** For the introducer needle, 0.2 mL of the USCA was used, and the needle was positioned horizontally throughout. In steps I–II, the inner stylet was removed from the outer cannula. In steps III–IV, the syringe was connected to the outer cannula. In steps V–VI, the outer cannula was flushed with the USCA, and the syringe was subsequently removed. In the final steps, VII–VIII, the inner stylet was put back again

The study conducted trials using two different types of biopsy needles and an introducer needle, as specified in Table 1, in a water bath phantom. The USCA sulfur hexafluoride (SonoVue; Bracco SpA, Milan, Italy) was used, both to prime the needles and to achieve a contrast-enhanced background in the phantom. The needles were primed as presented in Fig. 1 and Online Resources 1–3 using a Luer slip syringe (1 ML 0,01 LUER TUBERCULIN, CODAN Medical Aps, Rødby, Denmark). Unprimed needles were used as controls.

**Table 1:** The three needle types and, where applicable, associated instruments used in the experiment

Needle type	Components	Description
Side-notch biopsy needle	Pro-Mag Ultra Automatic Biopsy Instrument with Pro-Mag Biopsy Needle 18 ga × 16 cm	Non-disposable fully automatic biopsy instrument with practically non-separable needle side-notch biopsy needle
Full core biopsy needle	BioPince Ultra Full Core Biopsy Instrument 18 ga × 20 cm	Disposable fully automatic biopsy instrument with non-separable full core biopsy needle
Introducer needle	Introducer Needle 17 ga × 16.8 cm	Coaxial introducer needle included in the BioPince set

All needles were from the same manufacturer (Argon Medical Devices, Inc., TX, U.S.A.)

To evaluate each needle type, 10 separate sets were observed, each consisting of 6 punctures divided into 3 matched pairs of one USCA-primed needle and one control. This resulted in a total of 30 pairs per needle type and an overall sample size of 180.

**Fig. 2:**
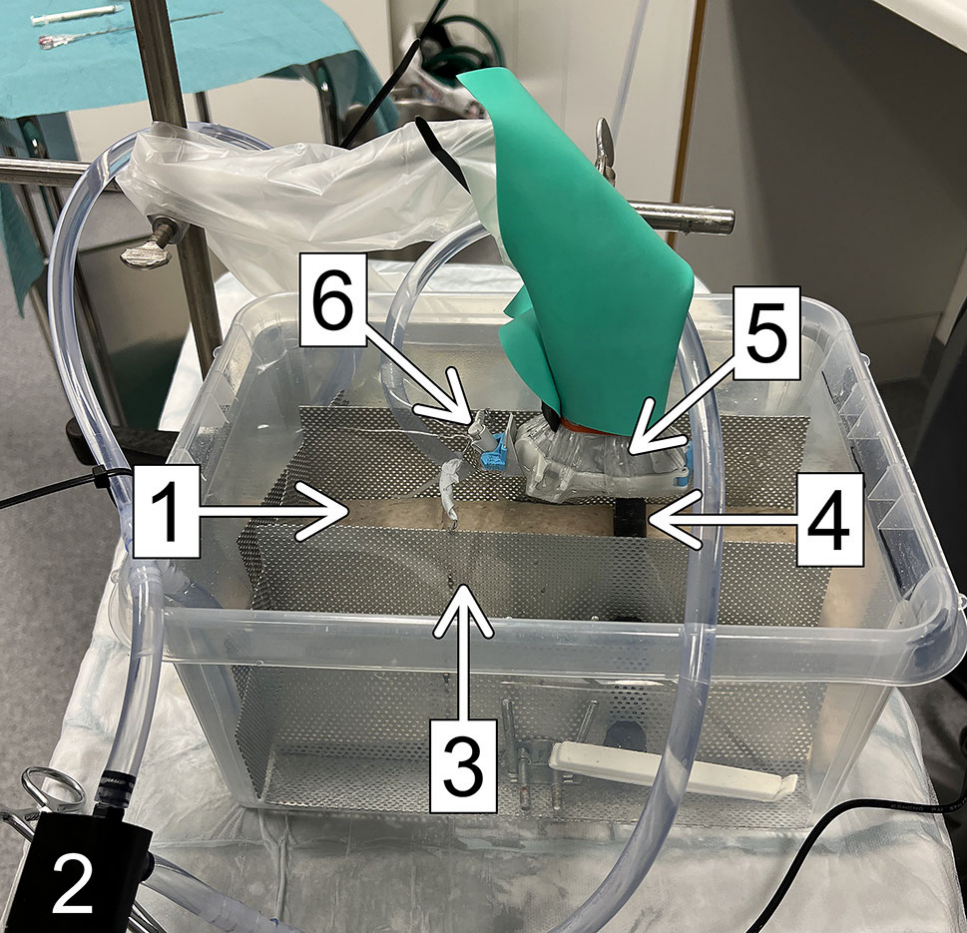
Phantom consisting of a plastic container filled with water (7 L) to which the USCA was added in a concentration resembling the human liver. 1: One cm thick slices of canned ham was placed at the bottom of the container to reduce the mirroring artifacts. 2: An electrical water pump was connected to the plastic tubes in the water, creating a circulating system of water to achieve a uniform contrast agent concentration. 3: Metal stand to keep the compounds in place. 4: Water filled polyolefin hose mimicking a tumor. 5: Transducer enclosed in a non-latex probe cover and set up on a metal tripod. 6: Needle guide pointing toward the polyolefin hose

The water bath phantom (depicted in Fig. 2) was set up as below:

- A plastic container was filled with 7 L of water, to which 0.3 mL of USCA was added to resemble a perfused human liver after intravenous USCA administration^1^
- The liquid was mixed around with an electric pump (240 L/h) and exchanged after every set of six punctures to retain a uniform background intensity
- One-cm-thick slices of canned ham (SPAM, Danish Crown UK Ltd., Manchester, UK) were placed at the bottom of the container to reduce any reflections
- A polyolefin hose (thickness 0.3 mm; diameter 12.7 mm) filled with water and with the ends closed was placed just above the canned ham to mimic a tumor that is not perfused (anechogenic)
- The canned ham and polyolefin hose were fixed by a self-made metal stand

Scanning was performed using an ACUSON Sequoia unit (Siemens Medical Solutions, Inc., Mountain View, CA, U.S.A.) with a 5C1 curved transducer. The transducer was enclosed in a non-latex probe cover and set up on a metal tripod. A needle guide (Verza, CIVCO Medical Instruments Co., Inc., IA, U.S.A.) in setting 3 (61°), with corresponding electronically generated guidance lines, was used. Imaging was performed with the following settings:

- Dual-screen mode with CEUS-mode image to the left (frequency setting low) and low MI B-mode image to the right (frequency setting mid)
- Maximum depth 9 cm, with the focus set in the deep portion of the image
- Gain standardized and set to −10 dB for the CEUS-mode image and 0 dB for the B-mode image
- Recording with 10 frames per second

Immediately after (approximately a few seconds) the priming, the needles were inserted via the needle guide until it just reached the polyolefin hose without deforming or penetrating it. The courses of the needles were recorded as video clips of approximately six seconds (3 s of needle insertion and 3 s at the needle’s maximum depth). The needles were subsequently retracted, and the biopsy instruments fired off outside the phantom. Thereafter, the needles were primed again before the next observation.

For both the side-notch and full core biopsy needle types, a new USCA primed biopsy needle was used per set (each needle was, thus, primed three consecutive times before being disposed). This procedure was applied to resemble clinical conditions and to evaluate the possible impact on needle visibility with repeated primings. The latter since there was a suspicion that repeated loading and firing of the biopsy instruments subsequently could make the USCA propagate toward the non-sharp end of the biopsy in the space between the inner stylet and the outer cannula, and thus possibly affect the needle visibility.

### Image Processing and Evaluation

**Fig. 3:**
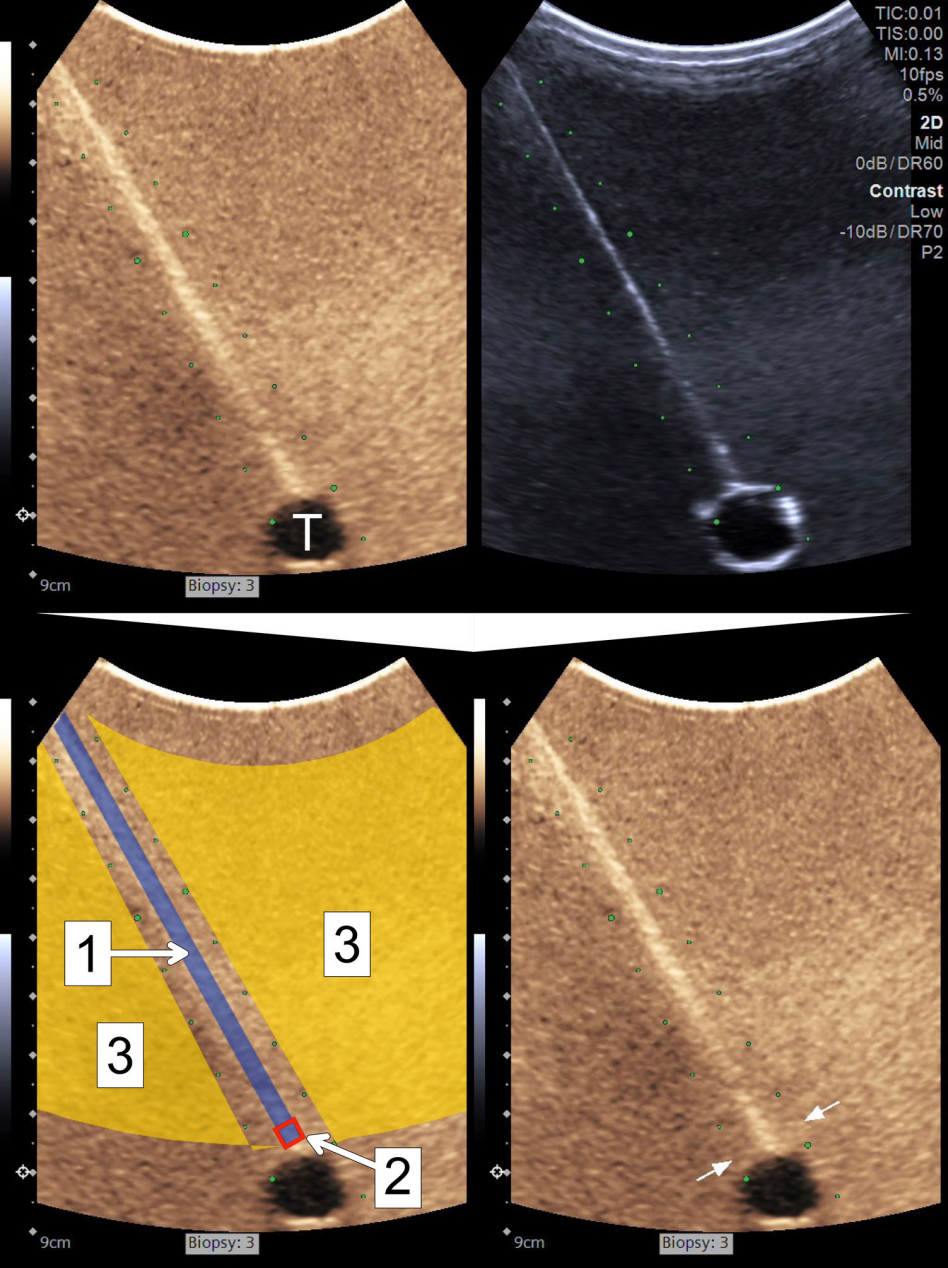
Top row: original dual-mode image with contrast-specific imaging-mode image to the left and low mechanical index B-mode image to the right, showing a full core needle primed with the ultrasound contrast agent in its end position, adjacent to the polyolefin hose serving as an echogenic, thus mimicking a tumor that is not perfused (T). Bottom row: Contrast-specific imaging-mode image from the top row (same cropped version both left and right). In the left, bottom row, image, the two regions of interest (ROIs) and the background have been colored and numbered from 1 to 3. 1: ROI representing the entire needle course (blue polygon with diameter of 3.5 mm). 2: ROI representing the needle tip (area within the red square with a side of 3.5 mm, adjacent to the simulated tumor). 3: Background (yellow polygon covering the area outside the electronically generated guidance lines with the same minimum and maximum depth as for the entire needle visibility). In the right bottom border, the depth of the needle tip has been pointed out by two arrows as it was presented for the assessors

The visibility of both the entire needles and the needle tips was evaluated both quantitatively and qualitatively. Analysis of the CEUS-mode visibility was performed. However, the analysis of the B-mode visibility was waived due to the inherent limitations related to using a water bath. The latter since good B-mode needle visibility is obtained in liquids despite low MI [9]. The B-mode image part of the recordings, however, was used to position the regions of interests (ROIs) correctly and to add arrows to clarify the end position of the needles for the assessors in the qualitative evaluation (see Fig. 3).

The recordings were processed with the application ImageJ version 1.53t (Wayne Rasband, National Institute of Health, Bethesda, MD, U.S.A.). Statistical analyses were performed with RStudio version 2022.12.0 + 353 software (RStudio, Inc., Boston, MA, U.S.A.). Statistical tests were 2 tailed and performed at a 0.05 significance level.

### Quantitative Evaluation

The needle visibility was quantitatively estimated by the contrast-to-noise ratio (CNR):

$$\mathrm{CNR}=\frac{|{\mu }_{i}-{\mu }_{o}|}{\sqrt{{{\sigma }_{i}}^{2}+{{\sigma }_{o}}^{2}}}$$

where *μ*_*i*_ is the mean signal intensity inside the ROI; *μ*_*o*_ is the mean signal intensity outside the ROI; *σ*_*i*_ is the variance of the intensity inside the ROI; and *σ*_*o*_ outside the ROI [14–16]. Two ROIs (entire needle and needle tip) and the area outside the ROIs (represented by a polygon at the same depth as the entire needle ROI) were defined in the CEUS-mode part of each recording, as illustrated in Fig. 3. The mean and standard deviation of the signal intensity were measured in the two ROIs as well as the background area for the image frames with the needles inserted in their end positions. CNRs were calculated for the entire needle and needle tip. The means of the CNRs were calculated for each puncture from which the medians were computed, and the differences in CNRs were tested with Wilcoxon signed rank (samples were matched for each of the pairs with the USCA versus the controls.)

For the two biopsy needles studied, the CNRs for the entire needles were calculated for each of the orders (first, second, and third) of the needle primings separately. This was done to test whether an improvement in the needle visibility could be achieved, independent of the three orders of primings.

### Qualitative Evaluation

**Fig. 4:**
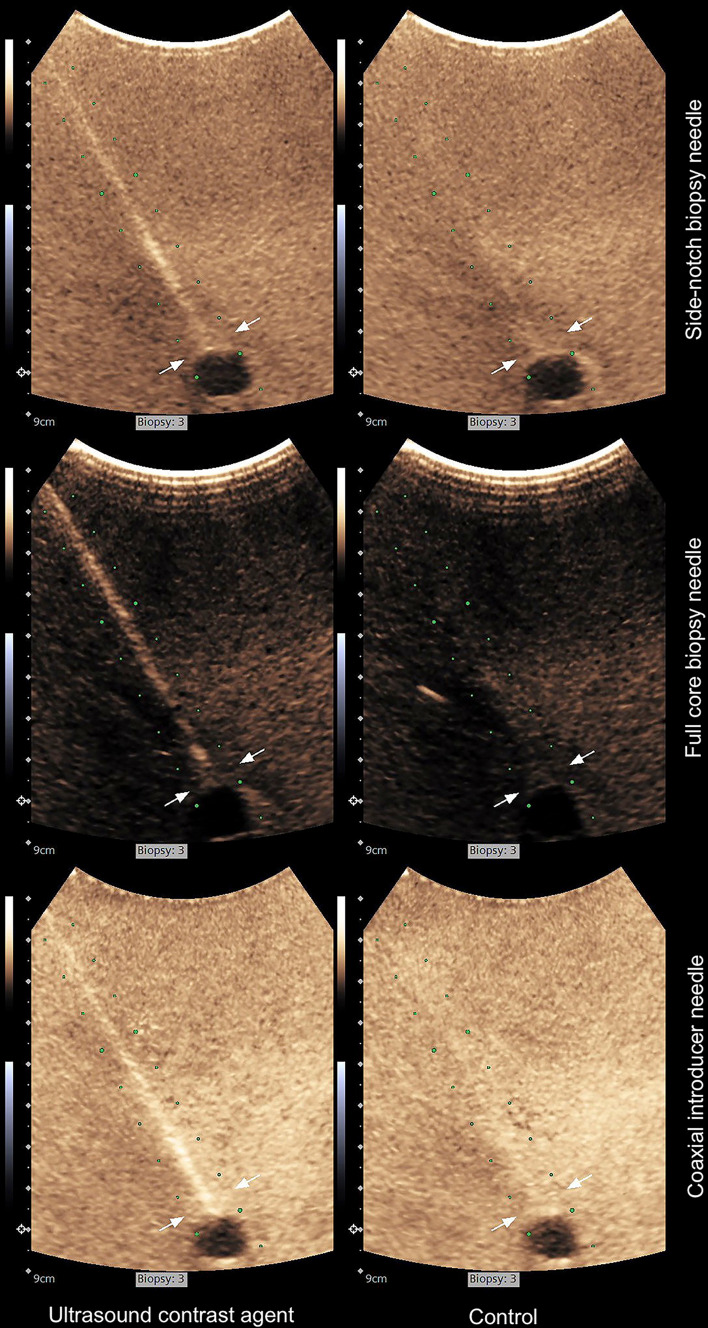
The first pairs of punctures (primed with ultrasound contrast agent versus controls) from the first of the three punctures in the first of the ten sets for the three needle types studied in the contrast-specific imaging-mode, as presented for the assessors. From the image frames with the needles in their end positions (end positions marked with white arrows), the median ones were selected for this set of images

Recordings, including both the needle insertion and the needle in the end position phase, were evaluated by three radiologists specialized in ultrasound (all with at least 20 years of experience) in a blinded manner. The radiologists were presented matched pairs of videos (USCA primed needle versus control) next to each other in random order via a web interface (see Fig. 4 and all 180 observations in Online Resource 4, respectively). The relative needle visibility was assessed by answering the following questions:

**The Needle in its Entirety:**

1. In which of the 2 videos is the entire needle most visible? (Video 1/Video 2)
2. Do you consider the difference in visibility to be clinically relevant? (Yes/No)

**The Needle Tip:**

1. In which of the 2 videos is the tip of the needle most visible? (Video 1/Video 2)
2. Do you consider the difference in visibility to be clinically relevant? (Yes/No)

The frequencies of the four possible combinations of the questions 1 and 2 were put together in 2 × 2 contingency tables. The assessors’ combined assessment, according to the majority principle, formed the primary outcome variable. Exact binominal tests (hypothesized probability of success of 0.5) were performed by comparing the frequency of USCA-primed needles with superior visibility and a clinically relevant difference in visibility with the total number of observations. The proportion of observations for which two or three of the assessors were in agreement was calculated.

## Results

### Quantitative Evaluation

**Fig. 5:**
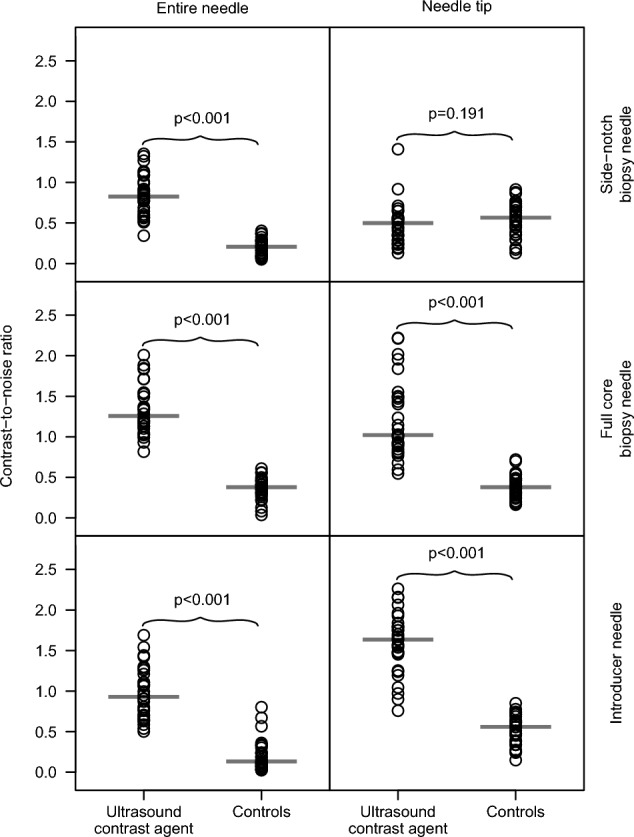
Contrast-to-noise ratio for ultrasound contrast agent primed needles versus controls for both the entire needles and the needle tips in the three different needle types studied (*n* = 180). The gray bars indicate the median values and the *p* values Wilcoxon signed-rank tests

The results of the quantitative evaluation are presented in Table 2 and Fig. 5. To summarize, after the USCA needle priming:

- The entire needle CNR was superior for all three needle types
- The needle tip CNR was superior for the full core biopsy needles and introducer needles, but equivalent to the controls for the side-notch biopsy needles
- The entire needle CNR for the two biopsy needles was superior regardless of the priming order (first, second, and third)

**Table 2:** Results of the quantitative evaluation

			Median CNR		
Needle	ROI	Order of priming	USCA	Controls	*p*^1^
Side-notch biopsy needle	Entire needle	1	0.8	0.19	0.002
		2	0.79	0.20	0.002
		3	0.94	0.21	0.002
		Aggregated^2^	0.83	0.21	< 0.001
	Needle tip	Aggregated^2^	0.50	0.57	0.191
Full core biopsy needle	Entire needle	1	1.21	0.38	0.002
		2	1.19	0.39	0.002
		3	1.43	0.35	0.002
		Aggregated^2^	1.26	0.38	< 0.001
	Needle tip	Aggregated^2^	1.02	0.38	< 0.001
Introducer needle	Entire needle	Aggregated^2^	0.93	0.13	< 0.001
	Needle tip	Aggregated^2^	1.63	0.56	< 0.001

*CNR* Contrast-to-noise ratio, *ROI* Region of interest, *USCA* Ultrasound contrast agent

^1^Wilcoxon signed-rank test comparing the USCA-primed needles with the controls

^2^Aggregation of the different order of primings (1–3)

### Qualitative Evaluation

The results of the qualitative evaluation are presented in Table 3. Overall, after the USCA needle priming, the needle was deemed more visible in a clinically relevant manner for all three investigated needle types for both the entire needle and the needle tip. This applied for the radiologists both independently and weighted together. Thereto, after the USCA needle priming, two of the three radiologists assessed the needles as being more visible in a clinically relevant manner in all observations (100% agreement) and when including all the three radiologists in 154 of the 180 assessments (86% agreement).

**Table 3:** Results of the qualitative evaluation

Side-notch biopsy needle									
*Entire needle visibility*									
Assessor		1		2		3		WT	
Filling		*USCA*	*Ctrls*	*USCA*	*Ctrls*	*USCA*	*Ctrls*	*USCA*	*Ctrls*
Clinically relevant	*Y*	25	0	30	0	30	0	30	0
	*N*	5	0	0	0	0	0	0	0
*p*^*1*^		< 0.001		< 0.001		< 0.001		< 0.001	
*Needle tip visibility*									
Assessor		1		2		3		WT	
Filling		*USCA*	*Ctrls*	*USCA*	*Ctrls*	*USCA*	*Ctrls*	*USCA*	*Ctrls*
Clinically relevant	*Y*	25	0	30	0	30	0	30	0
	*N*	5	0	0	0	0	0	0	0
*p*^*1*^		< 0.001		< 0.001		< 0.001		< 0.001	

Full core biopsy needle									
*Entire needle visibility*									
Assessor		1		2		3		WT	
Filling		*USCA*	*Ctrls*	*USCA*	*Ctrls*	*USCA*	*Ctrls*	*USCA*	*Ctrls*
Clinically relevant	*Y*	23	0	30	0	30	0	30	0
	*N*	7	0	0	0	0	0	0	0
*p*^*1*^		0.005		< 0.001		< 0.001		< 0.001	
*Needle tip visibility*									
Assessor		1		2		3		WT	
Filling		*USCA*	*Ctrls*	*USCA*	*Ctrls*	*USCA*	*Ctrls*	*USCA*	*Ctrls*
Clinically relevant	*Y*	24	0	30	0	30	0	30	0
	*N*	6	0	0	0	0	0	0	0
*p*^*1*^		0.001		< 0.001		< 0.001		< 0.001	

Introducer needle									
*Entire needle visibility*									
Assessor		1		2		3		WT	
Filling		*USCA*	*Ctrls*	*USCA*	*Ctrls*	*USCA*	*Ctrls*	*USCA*	*Ctrls*
Clinically relevant	*Y*	29	0	30	0	30	0	30	0
	*N*	1	0	0	0	0	0	0	0
*p*^*1*^		< 0.001		< 0.001		< 0.001		< 0.001	
*Needle tip visibility*									
Assessor		1		2		3		WT	
Filling		*USCA*	*Ctrls*	*USCA*	*Ctrls*	*USCA*	*Ctrls*	*USCA*	*Ctrls*
Clinically relevant	*Y*	28	1	30	0	30	0	30	0
	*N*	1	0	0	0	0	0	0	0
*p*^*1*^		< 0.001		< 0.001		< 0.001		< 0.001	

*WT* Weighted together (by majority), *USCA* Ultrasound contrast agent, *Ctrls* Controls, *Y* Yes, *N* No

^1^Exact binominal tests (hypothesized probability of success of 0.5) by comparing the frequency of USCA-primed needles with superior visibility and a clinically relevant difference in visibility with the total number of observations

## Discussion

In this phantom model study, the described methods of USCA priming of three different needle types increased the needle visibility in all but one of the quantitative evaluations and in all qualitative evaluations performed. The quantitative evaluation of the side-notch biopsy needle tips constituted the only exception, with equivocal CNR compared to the controls. Furthermore, for both the biopsy needles studied, an increase in the entire needle visibility, in terms of CNR, was obtained after the USCA priming, independent of the orders of primings (first, second, and third). The latter indicated that the multiple rounds of loading and firing of the biopsy instruments associated with repeated priming were not required for the methods to work.

The concept of needle priming with USCA to improve the needle visibility in CEUS-mode has earlier been demonstrated ex vivo and in a water bath phantom model for a reusable biopsy instrument with disposable needles [10]. In contrast to the previously described USCA needle priming method, the current evaluated methods were performed without separating the needle components and did not require a temporary sharps holder. The currently described priming methods were thus simpler and more versatile by enabling a priming of disposable (i.e., exclusively non-separable) biopsy instruments and also introducer needles. Moreover, the USCA, instead of oxygen bubbles, was used in the water bath phantom; also, the qualitative evaluation form was modified to simplify the outcome measures and emphasize the clinical relevance.

In a letter to the editor, Chandrashekharaa et al. presented a case with satisfactory CEUS-mode biopsy needle visibility by using the USCA needle priming [11]. Their priming method was neither described in detail nor evaluated systematically and thereto required a larger volume of USCA (1–2 mL). However, the findings are promising for assessing the value of the USCA needle priming concept in vivo.

The lack of quantitatively increased needle tip visibility for the side-notch biopsy needle may be attributed to its larger proportion of inner stylet not being covered by the outer cannula at the tip (4.6 mm completely or partially not covered compared with 3.4 mm for the full core biopsy needle and 2.6 mm for the introducer needle). If this is considered, the position of the needle tip should still make it possible to estimate with a reasonable certainty in a clinical context.

Our study was limited to one of each of the three needle types included, one ultrasound machine, and one transducer, with the puncture angle and depth fixed. Furthermore, using a water bath phantom, despite the contrast-enhanced background used, is not exactly comparable to a clinical setting. Nevertheless, it was possible to distinguish between needles primed with an undiluted contrast agent and one primed with a contrast-enhanced background. Another limitation of using a water bath phantom is the inability to evaluate the B-mode needle visibility meaningfully due to the inherent good B-mode visibility in liquids [9]. Taken together, this underscores the need for a follow-up in vivo study of the described priming methods, including an evaluation of both the CEUS-mode and B-mode visibility.

## Conclusion

The in vitro CEUS-mode visibility of the biopsy and introducer needles was improved by using the described methods of needle priming with small amounts of USCA. The priming methods may be valuable in situations of poor needle visibility when performing biopsies in CEUS-mode, but the results need to be confirmed in vivo.

## Supplementary Information

Below is the link to the electronic supplementary material.

Supplementary file1 (MP4 66602 KB)

Supplementary file2 (MP4 124894 KB)

Supplementary file3 (MP4 99817 KB)

Supplementary file4 (PDF 14449 KB)

## Footnotes

1 The volume of the USCA was chosen based on the assumptions of an intravenous dose of 1.5 mL USCA and the liver containing approximately 14% of the total blood volume of 5 L in an adult [12, 13]
