# Supplementary material for: Ultrasound Contrast Agent Priming of Biopsy and Introducer Needles by Using a Small Syringe to Improve Needle Visibility in a Phantom Model
Source: Cardiovasc Intervent Radiol. 2023 Jul 12;46(8):1066–75. doi: 10.1007/s00270-023-03500-3 (PMC10382408; doi:10.1007/s00270-023-03500-3)
Supplement: Supplementary file 4 — Supplementary file4 (PDF 14449 KB) [file 270_2023_3500_MOESM4_ESM.pdf]

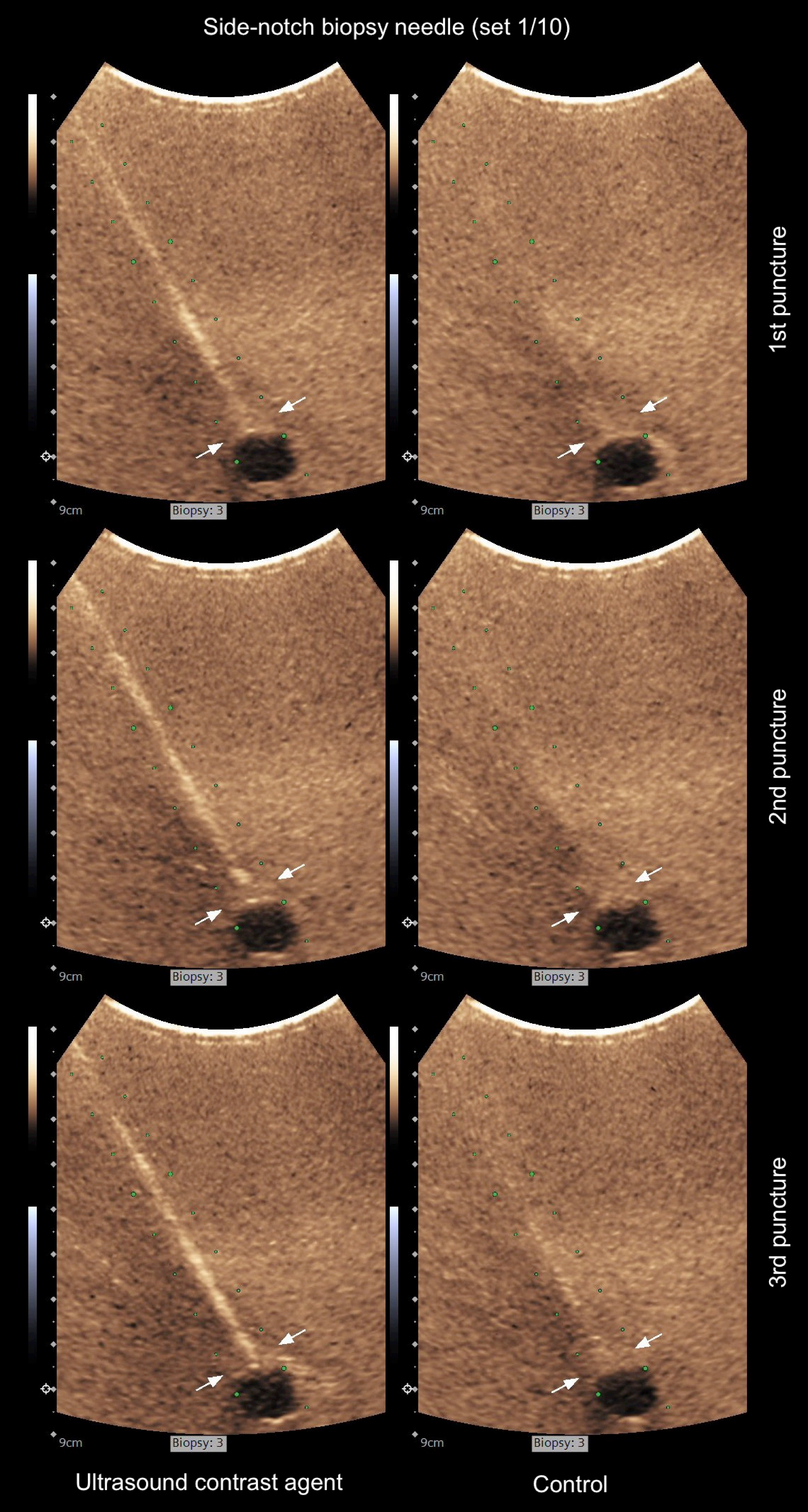

Side-notch biopsy needle (set 1/10)
1st puncture
2nd puncture
3rd puncture
9cm
Biopsy: 3
Ultrasound contrast agent
Control

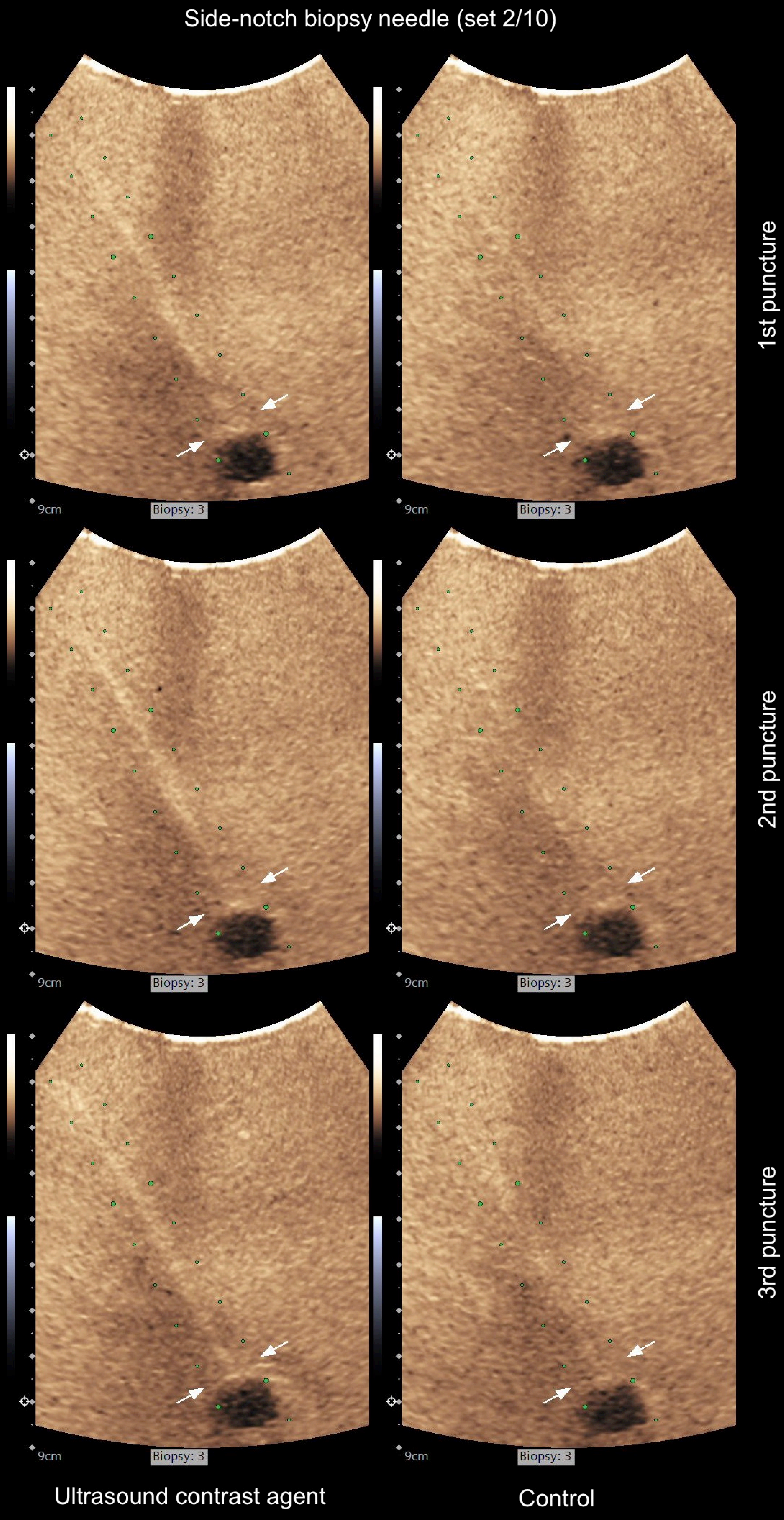
Side-notch biopsy needle (set 2/10)
1st puncture
2nd puncture
3rd puncture
9cm
Biopsy: 3
Ultrasound contrast agent
Control

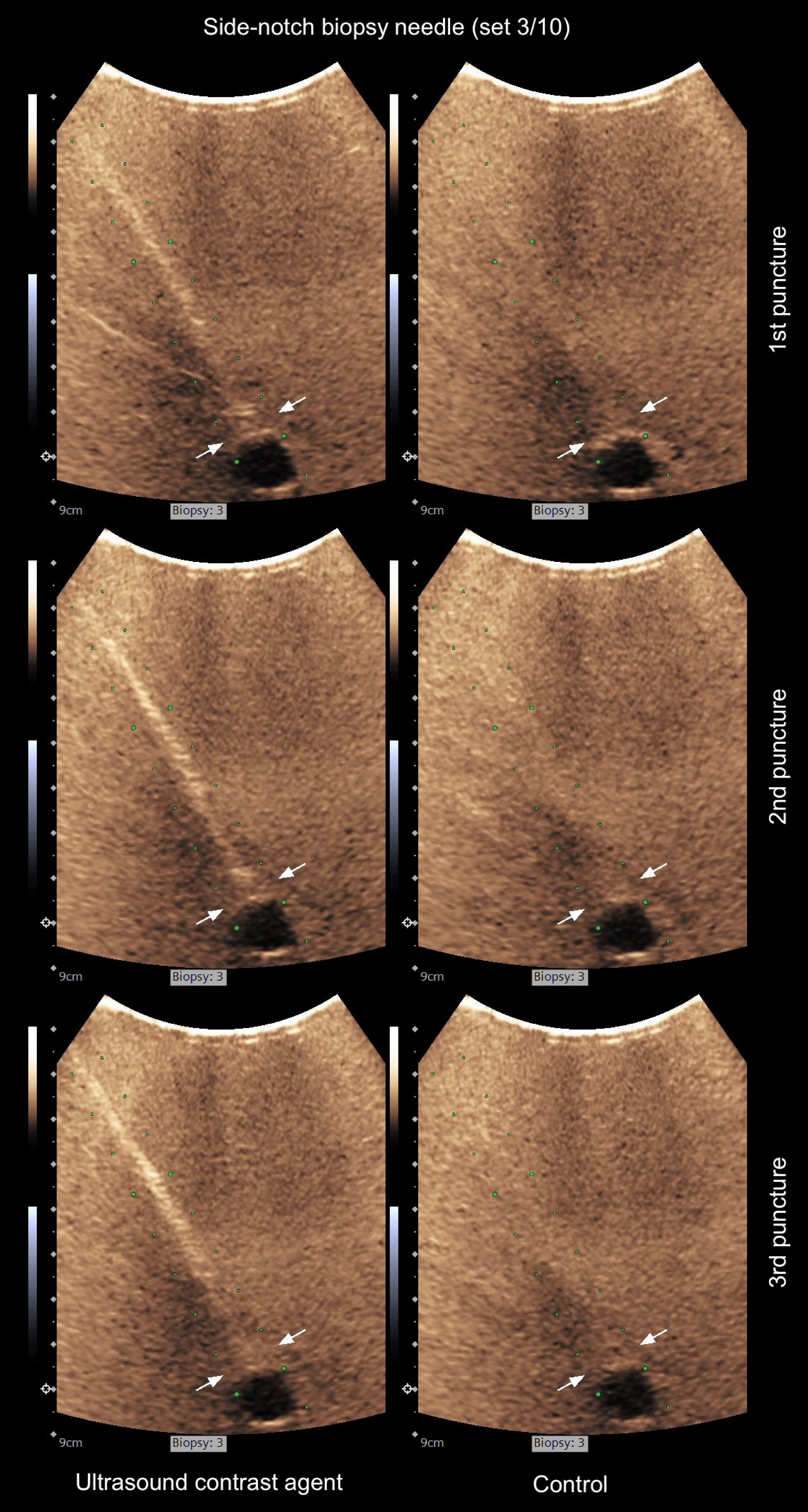
Side-notch biopsy needle (set 3/10)
1st puncture
2nd puncture
3rd puncture
9cm
Biopsy: 3
Ultrasound contrast agent
Control

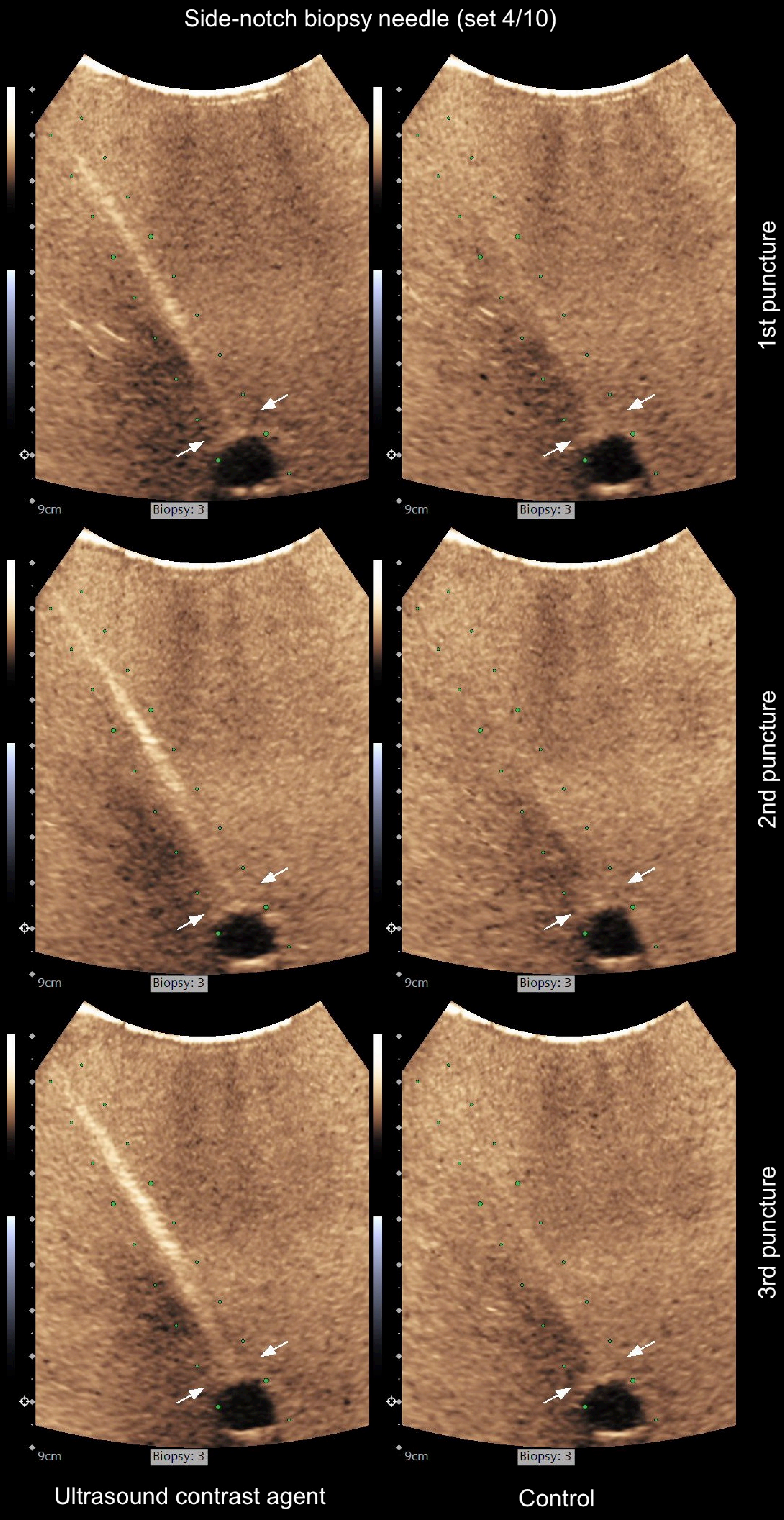
Side-notch biopsy needle (set 4/10)
1st puncture
2nd puncture
3rd puncture
9cm
Biopsy: 3
Ultrasound contrast agent
Control

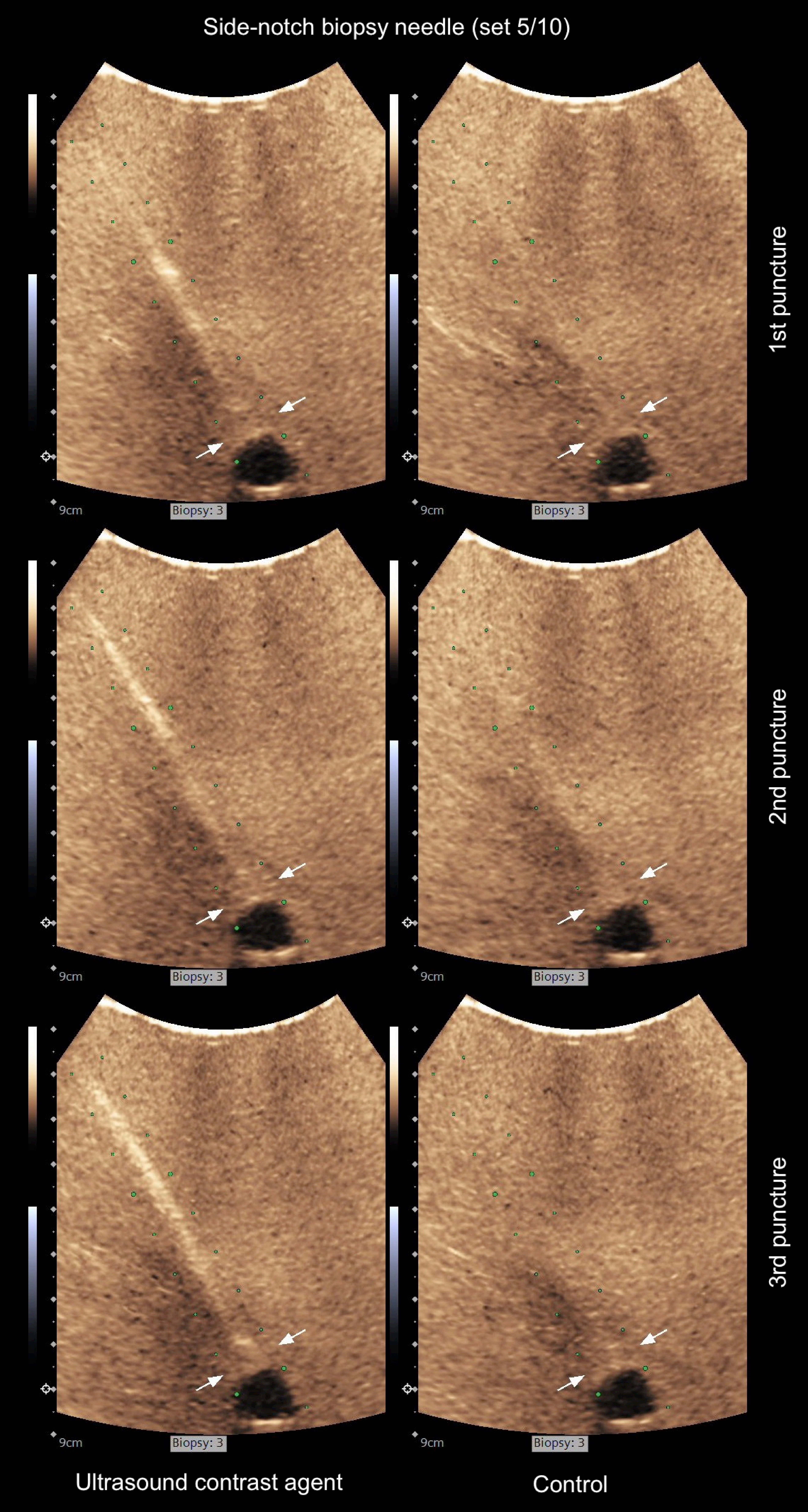
Side-notch biopsy needle (set 5/10)
1st puncture
2nd puncture
3rd puncture
9cm
Biopsy: 3
Ultrasound contrast agent
Control

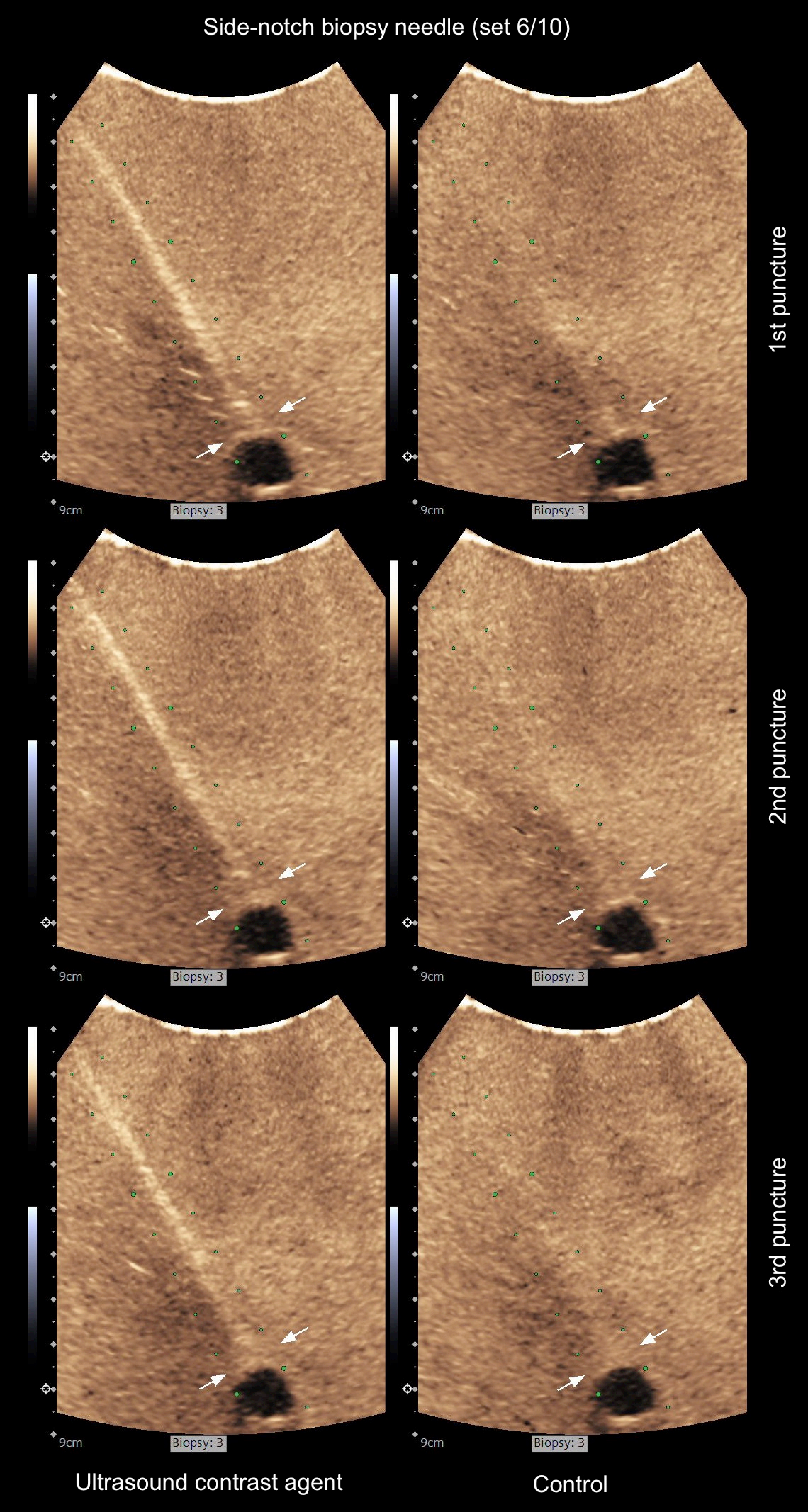

Side-notch biopsy needle (set 6/10)
1st puncture
2nd puncture
3rd puncture
9cm
Biopsy: 3
Ultrasound contrast agent
Control

Side-notch biopsy needle (set 7/10)

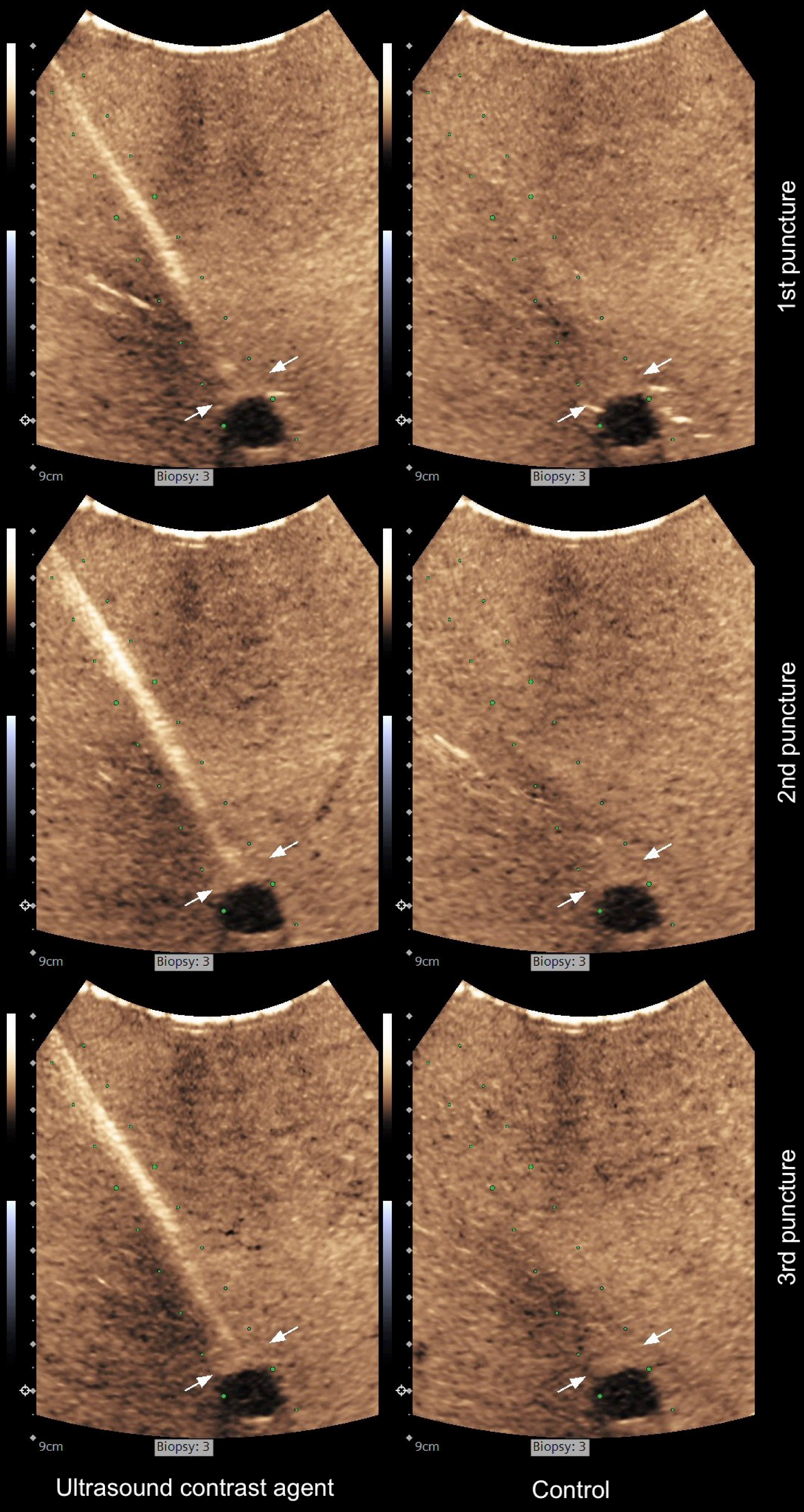

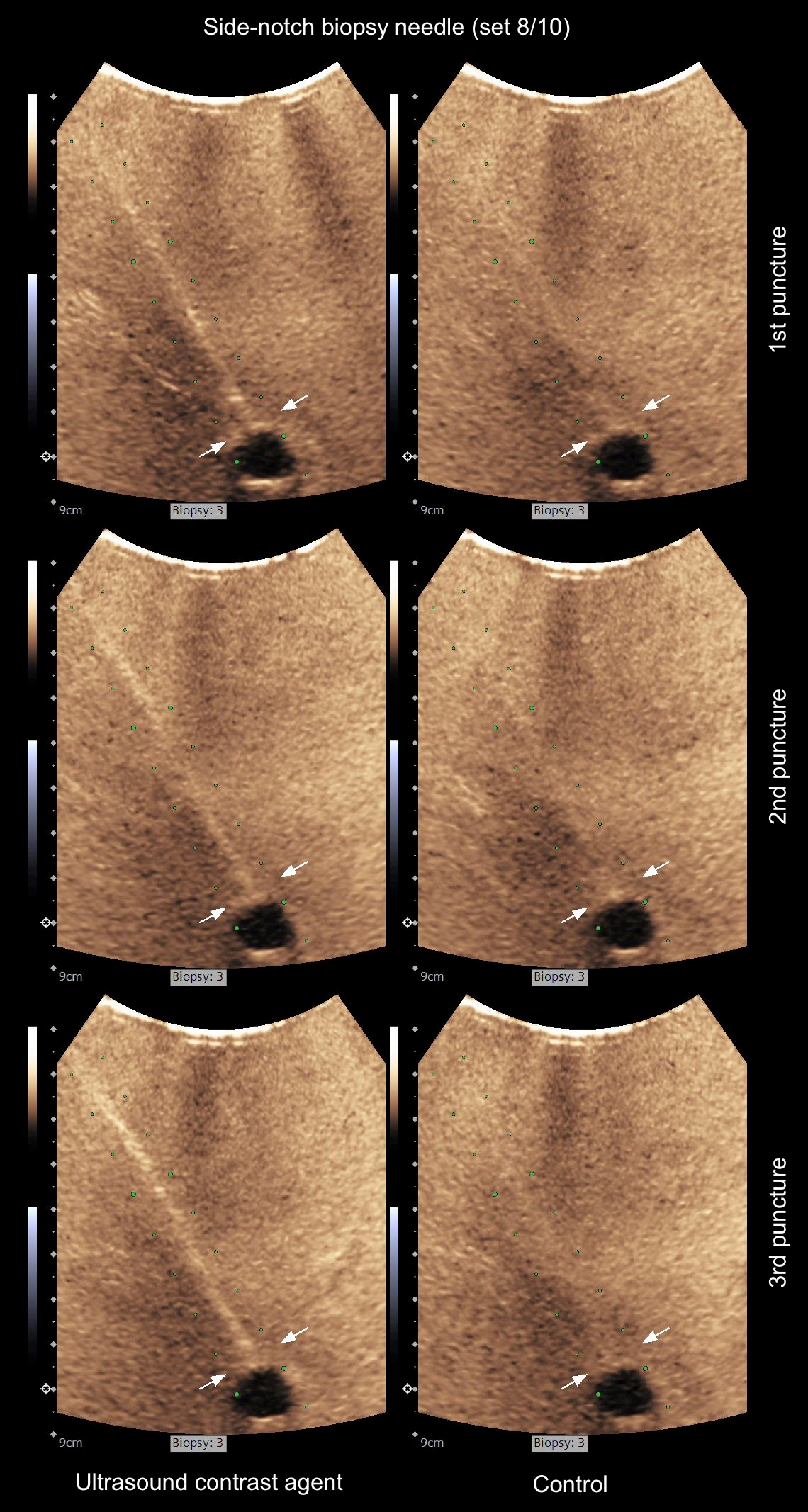
Side-notch biopsy needle (set 8/10)
1st puncture
2nd puncture
3rd puncture
9cm
Biopsy: 3
Ultrasound contrast agent
Control

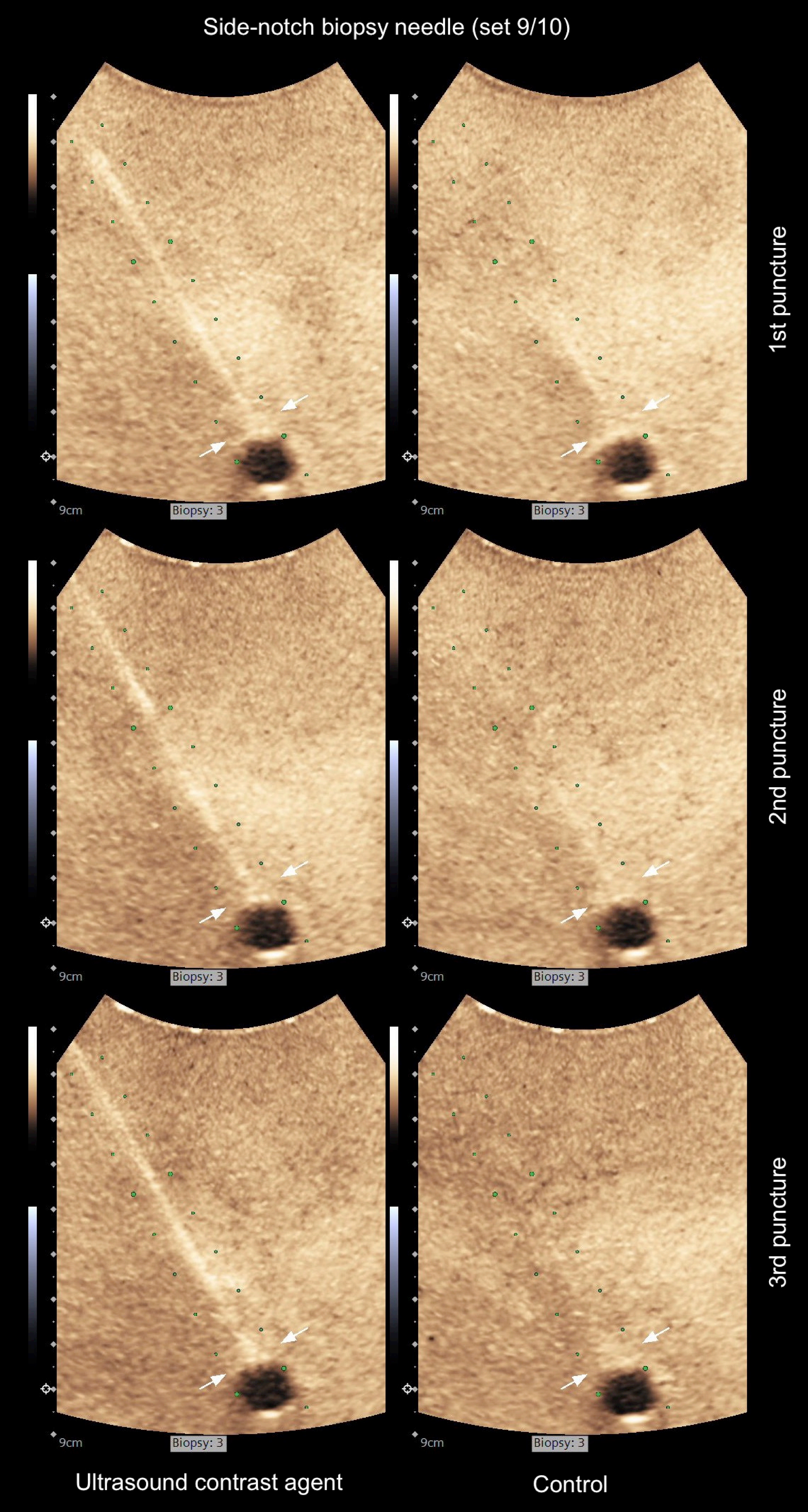

Side-notch biopsy needle (set 9/10)
1st puncture
2nd puncture
3rd puncture
9cm
Biopsy: 3
Ultrasound contrast agent
Control

Side-notch biopsy needle (set 10/10)

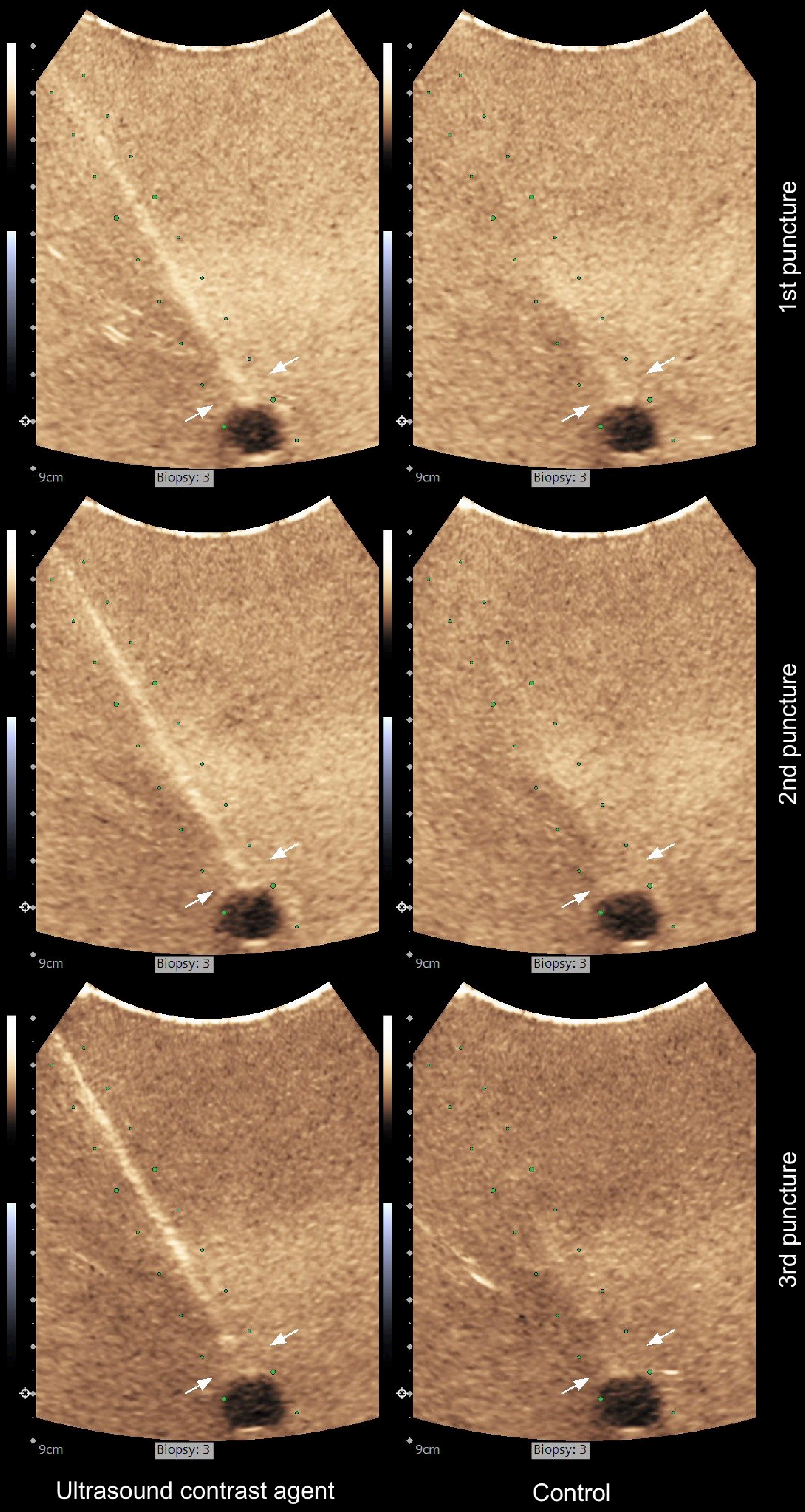

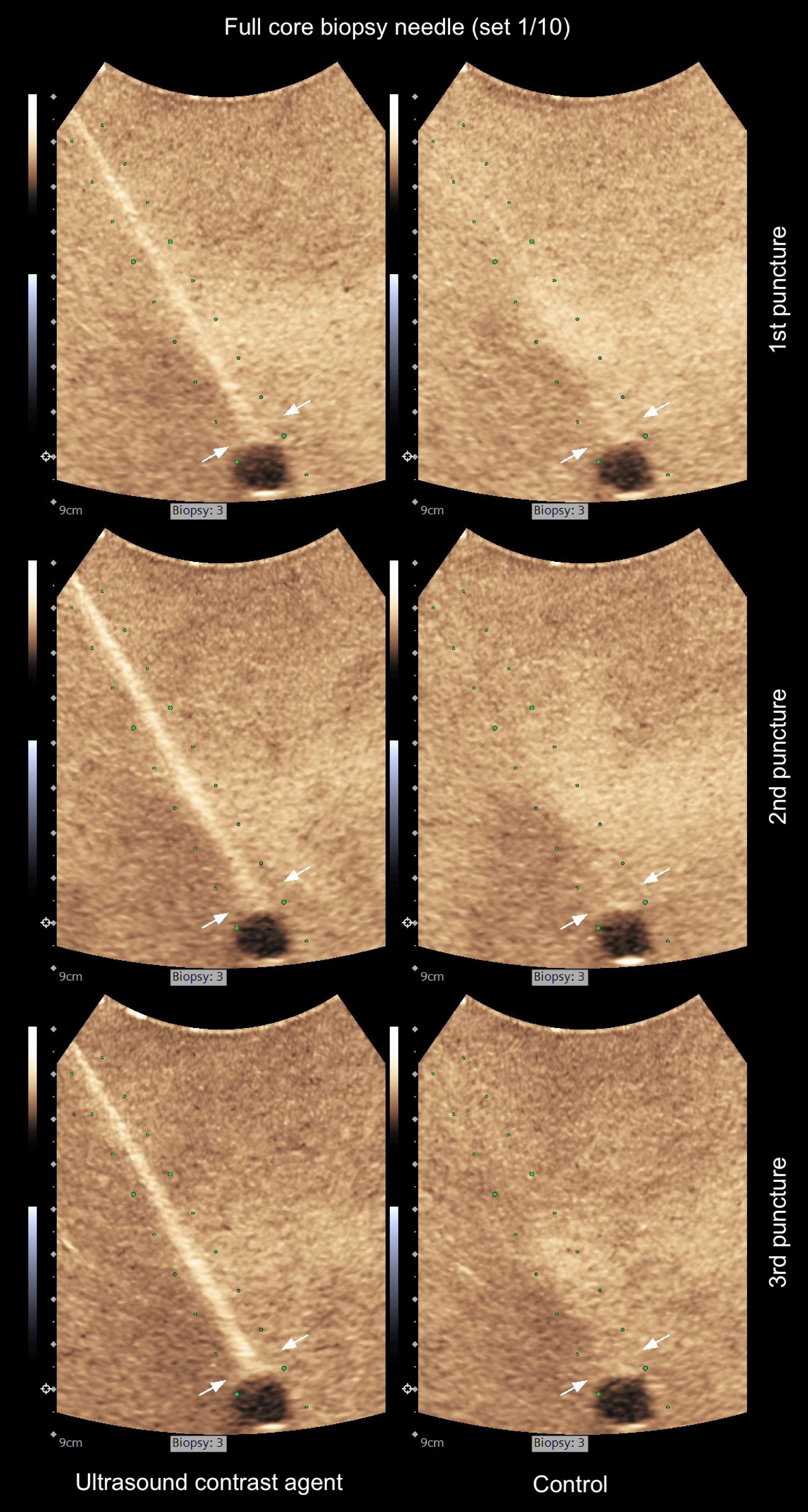
Full core biopsy needle (set 1/10)
1st puncture
2nd puncture
3rd puncture
9cm
Biopsy: 3
Ultrasound contrast agent
Control

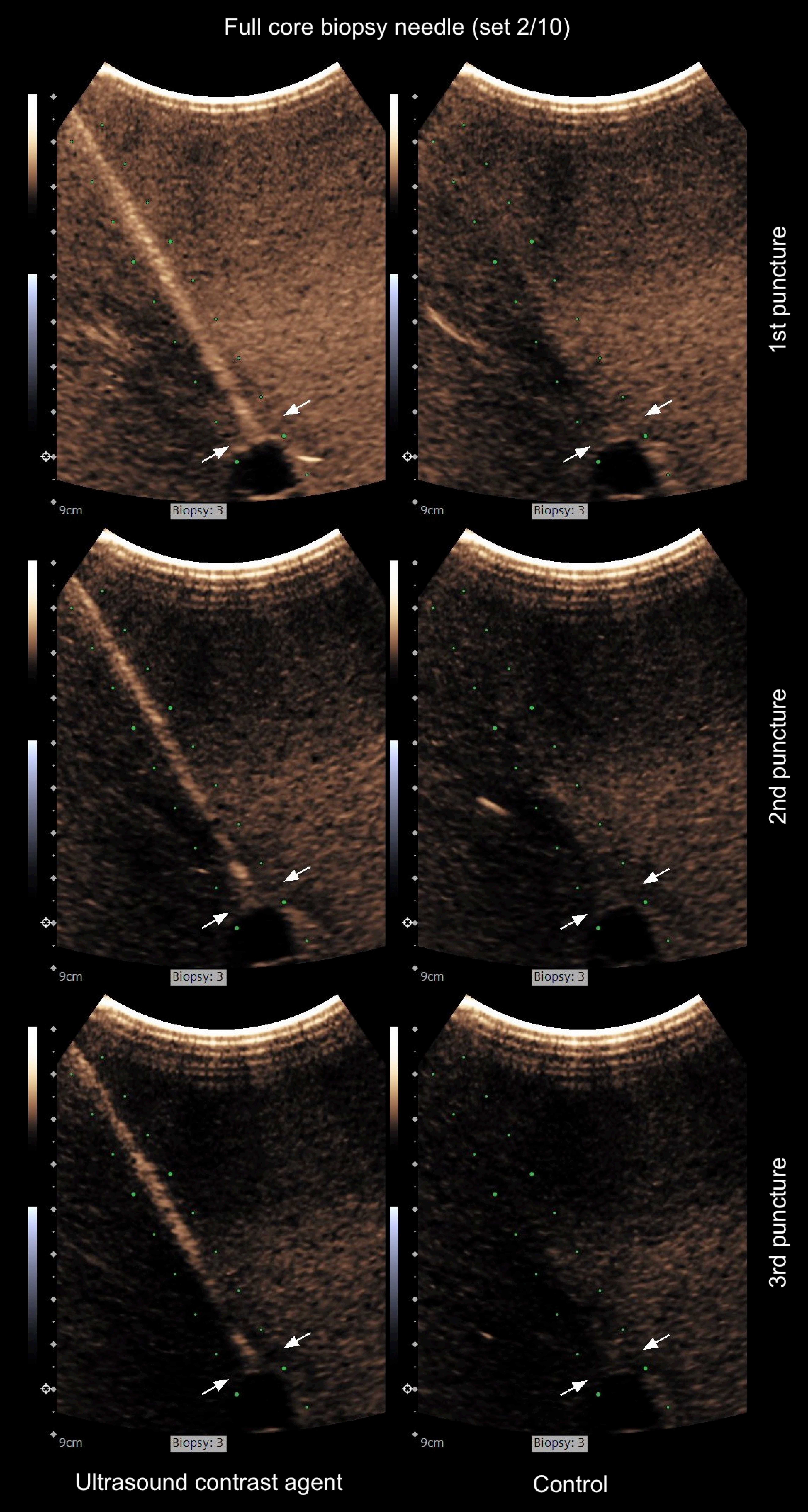

Full core biopsy needle (set 2/10)
1st puncture
2nd puncture
3rd puncture
9cm
Biopsy: 3
Ultrasound contrast agent
Control

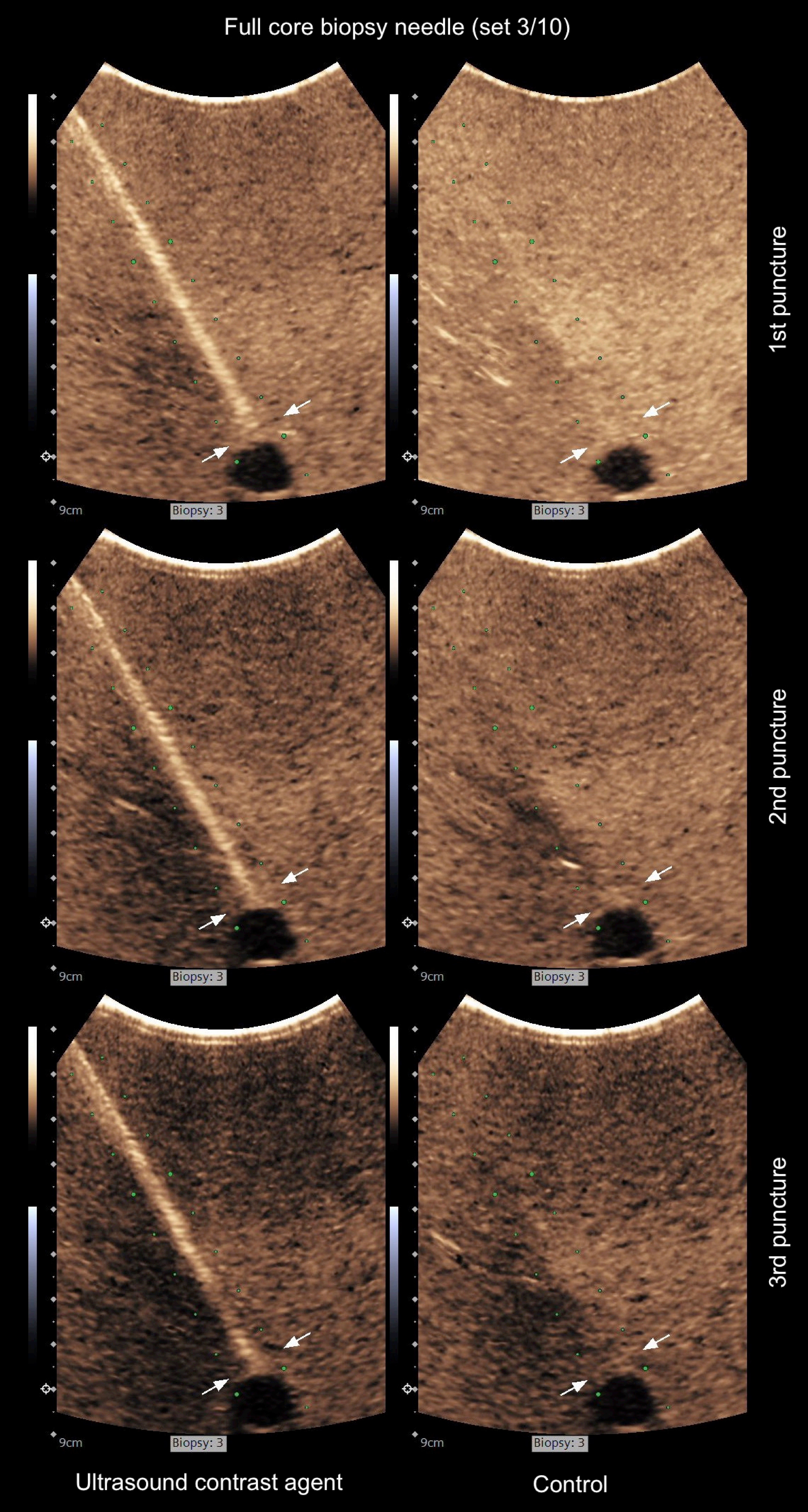
Full core biopsy needle (set 3/10)
1st puncture
2nd puncture
3rd puncture
9cm
Biopsy: 3
Ultrasound contrast agent
Control

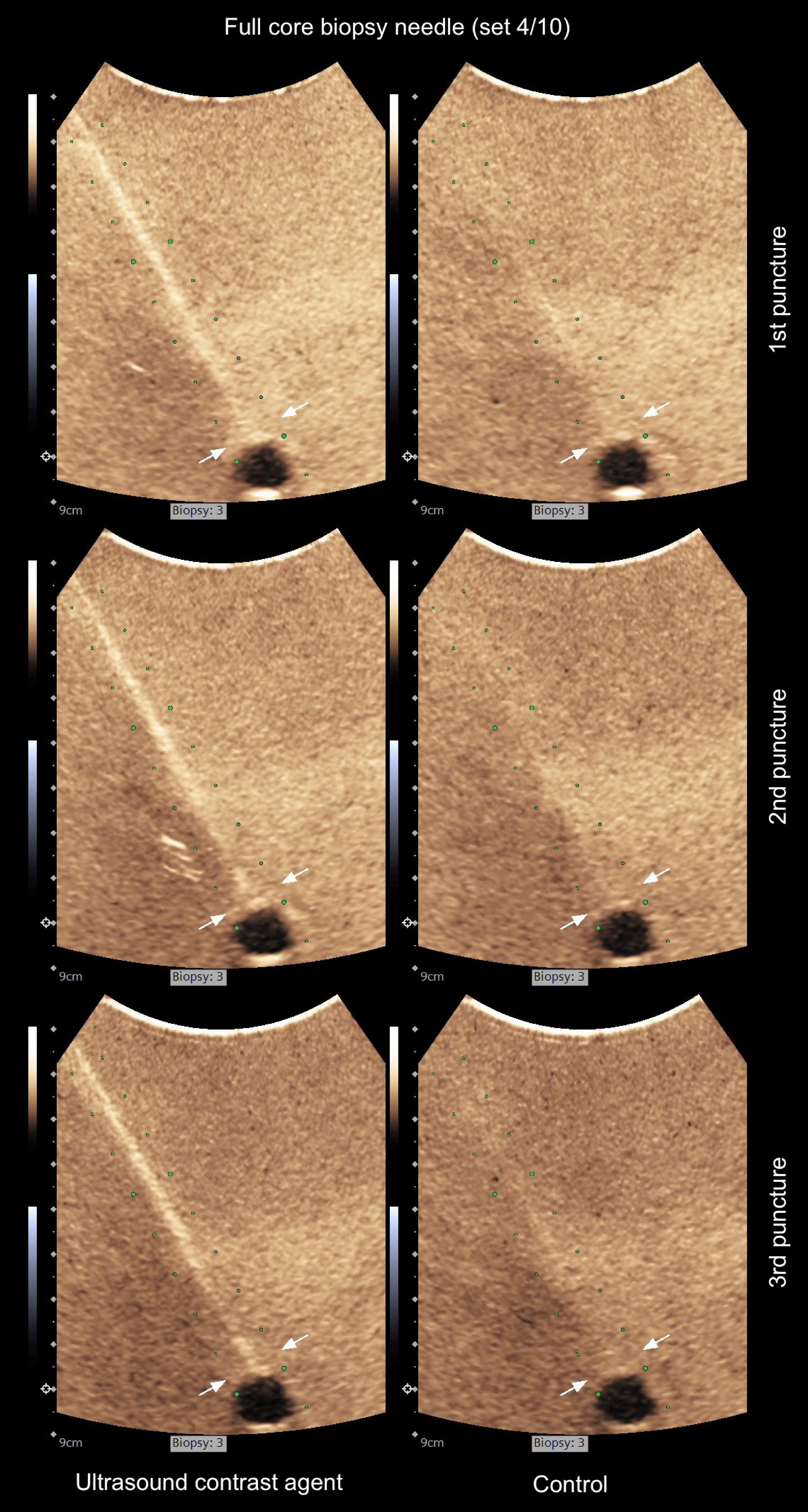
Full core biopsy needle (set 4/10)
1st puncture
2nd puncture
3rd puncture
9cm
Biopsy: 3
Ultrasound contrast agent
Control

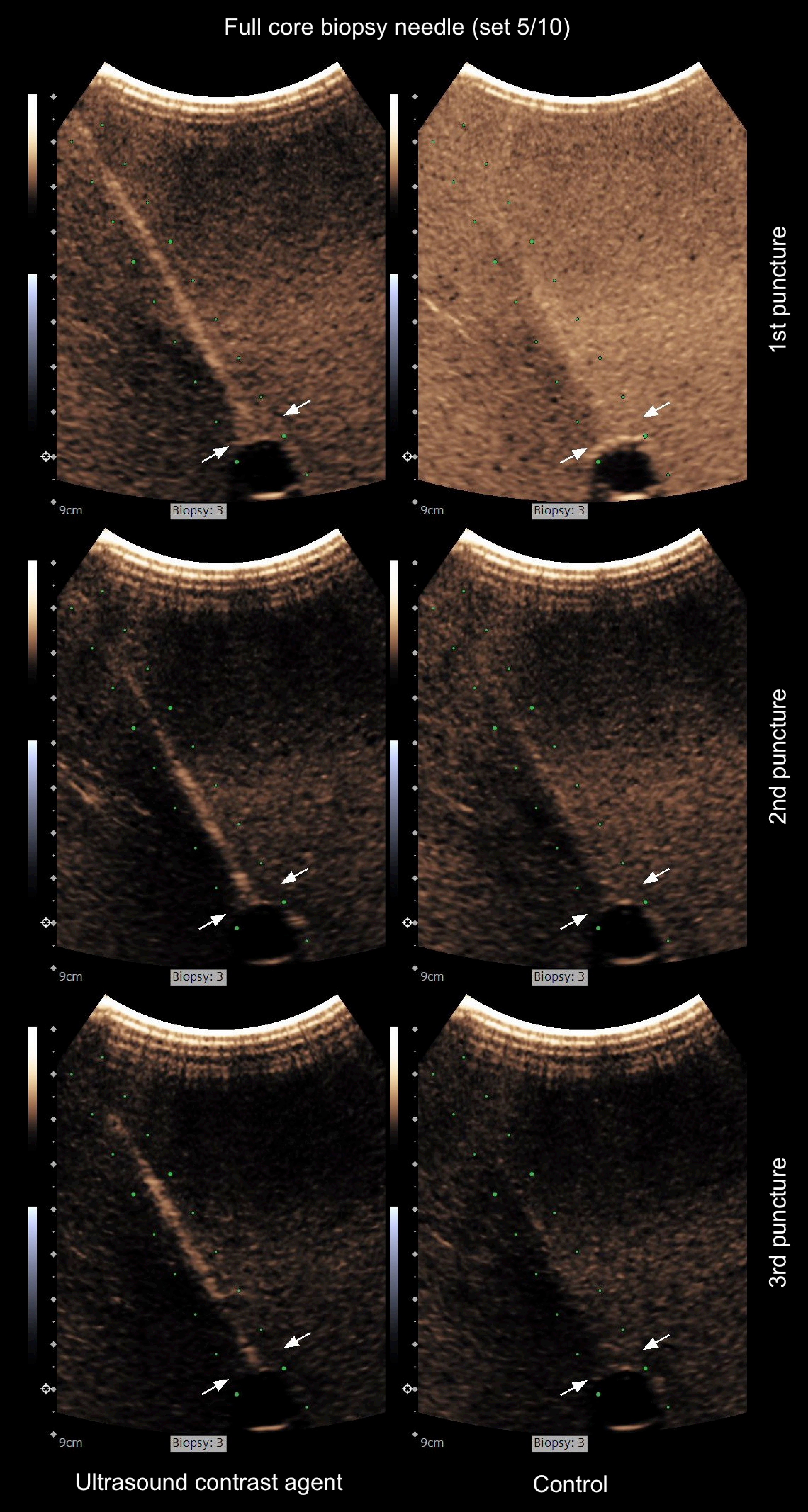

Full core biopsy needle (set 5/10)
1st puncture
2nd puncture
3rd puncture
9cm
Biopsy: 3
Ultrasound contrast agent
Control

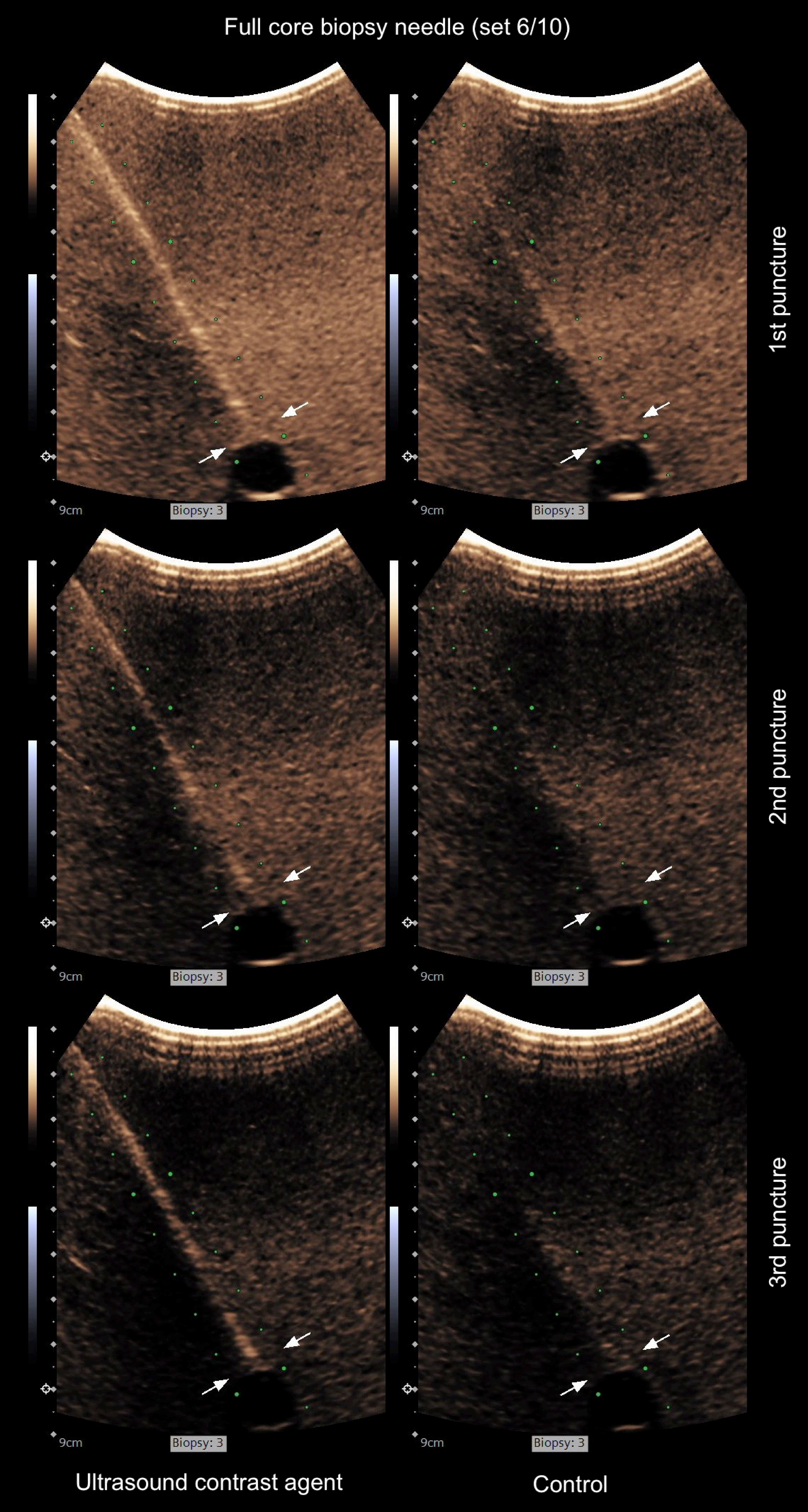
Full core biopsy needle (set 6/10)
1st puncture
2nd puncture
3rd puncture
9cm
Biopsy: 3
Ultrasound contrast agent
Control

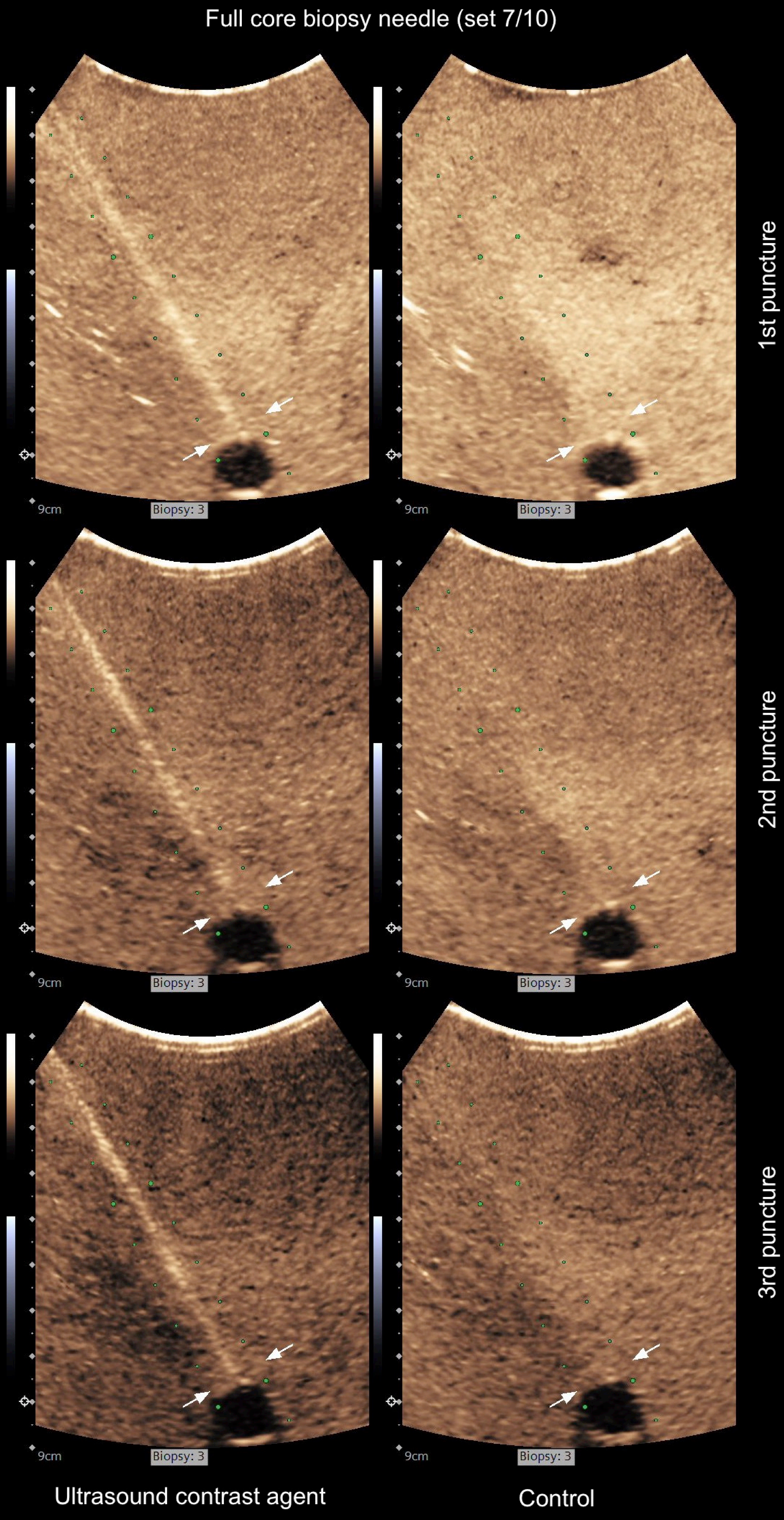
Full core biopsy needle (set 7/10)
1st puncture
2nd puncture
3rd puncture
9cm
Biopsy: 3
Ultrasound contrast agent
Control

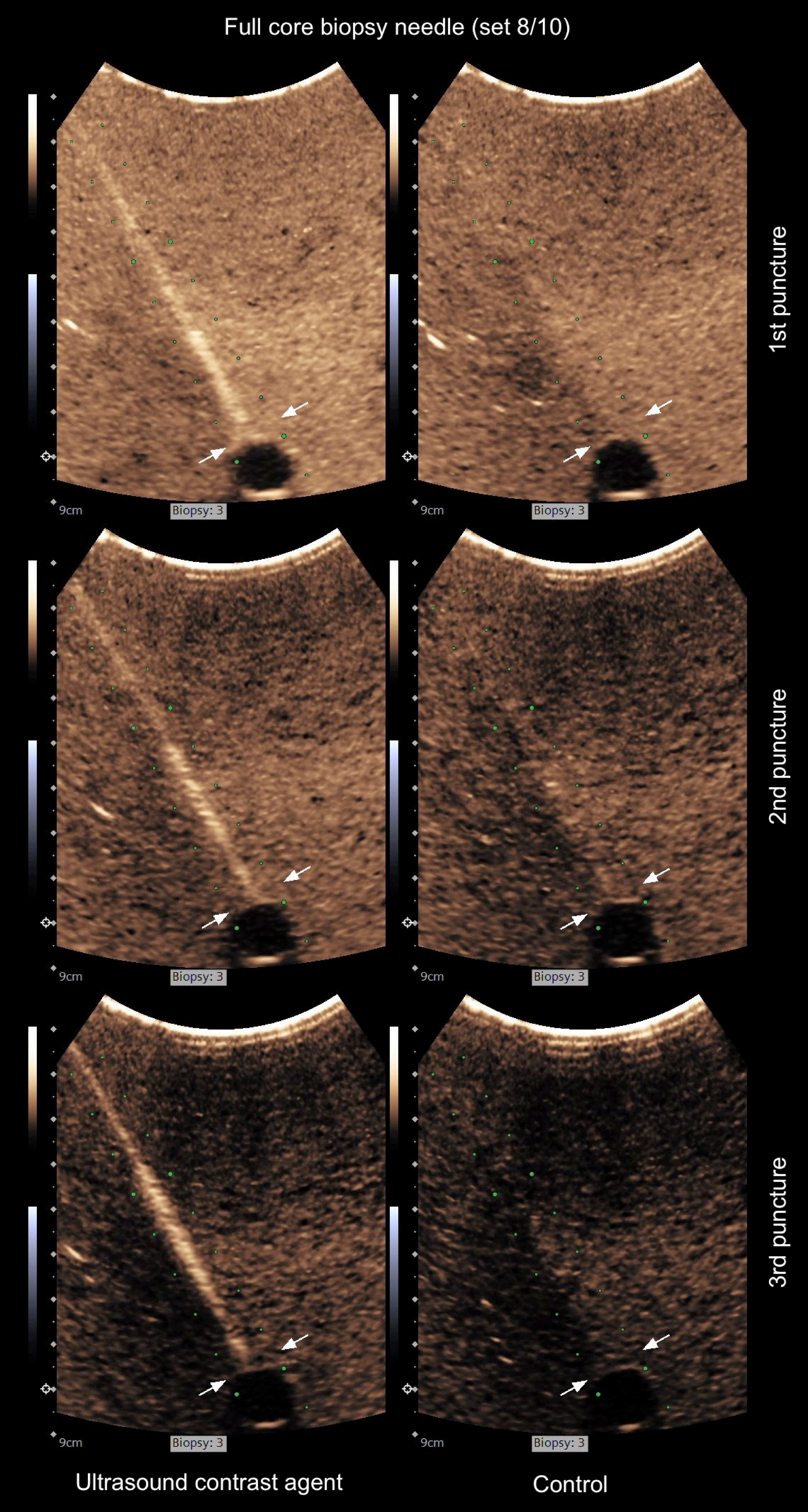
Full core biopsy needle (set 8/10)
1st puncture
2nd puncture
3rd puncture
9cm
Biopsy: 3
Ultrasound contrast agent
Control

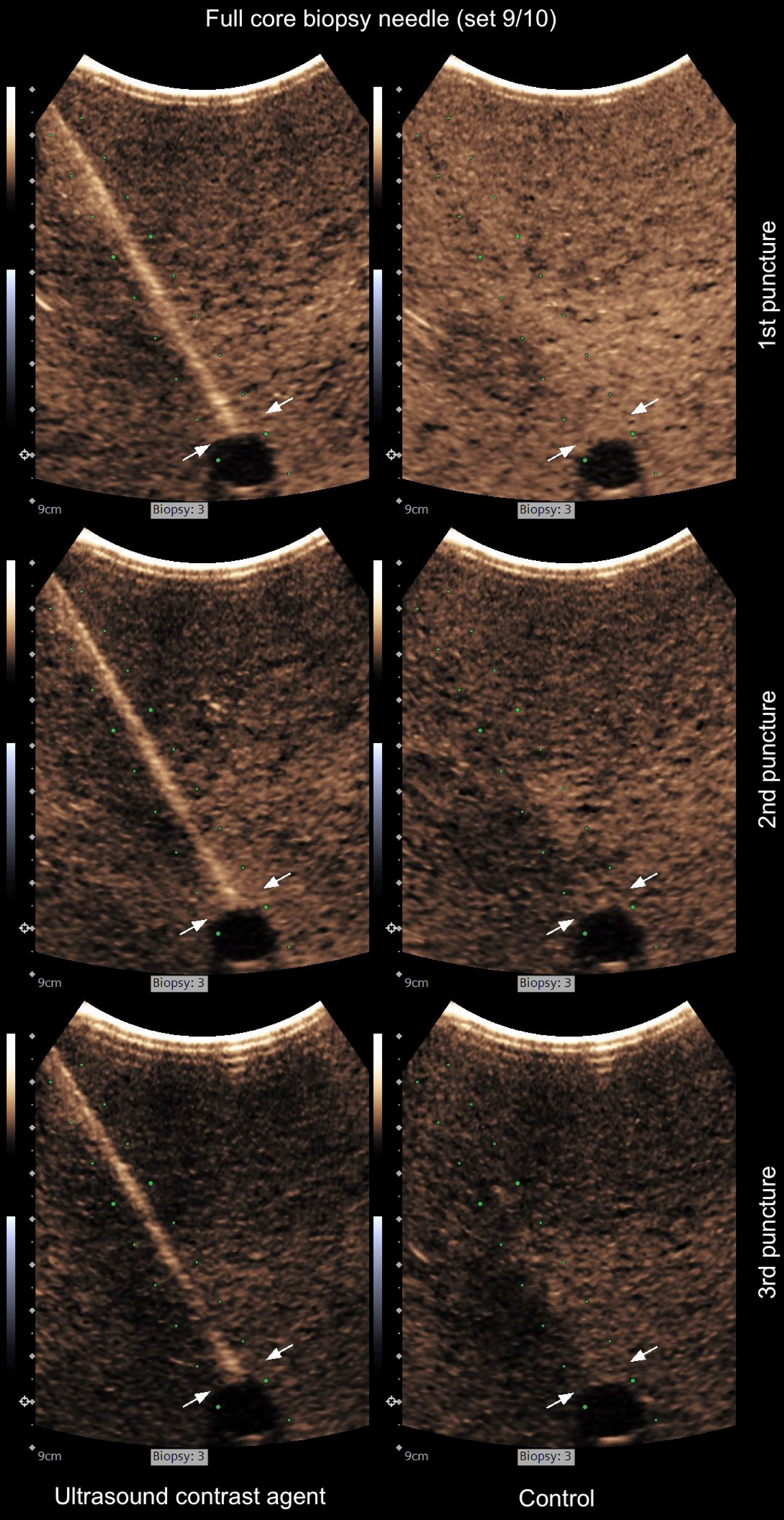

Full core biopsy needle (set 9/10)
1st puncture
2nd puncture
3rd puncture
9cm
Biopsy: 3
Ultrasound contrast agent
Control

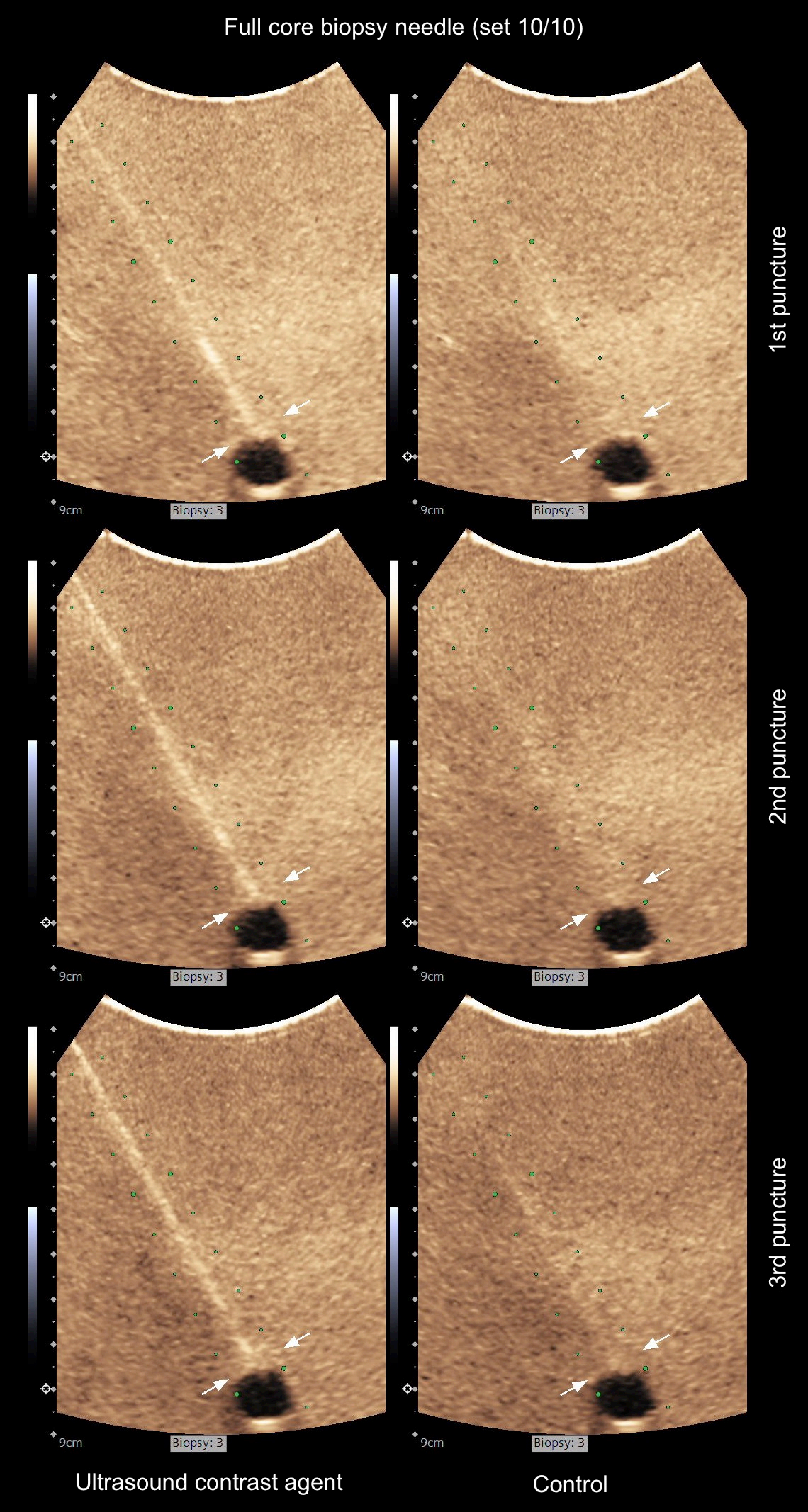
Full core biopsy needle (set 10/10)
1st puncture
2nd puncture
3rd puncture
9cm
Biopsy: 3
Ultrasound contrast agent
Control

Introducer needle (set 1/10)

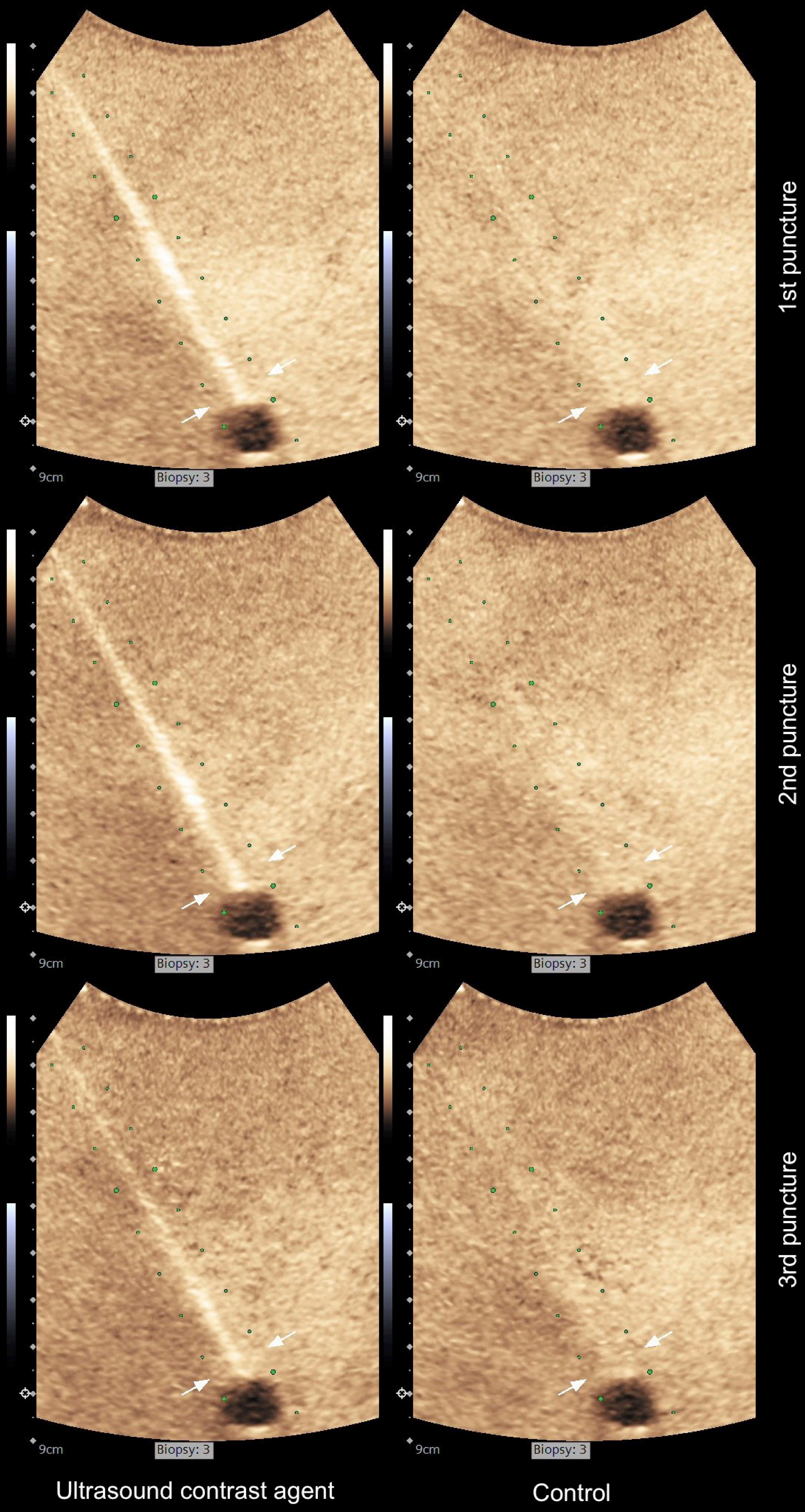

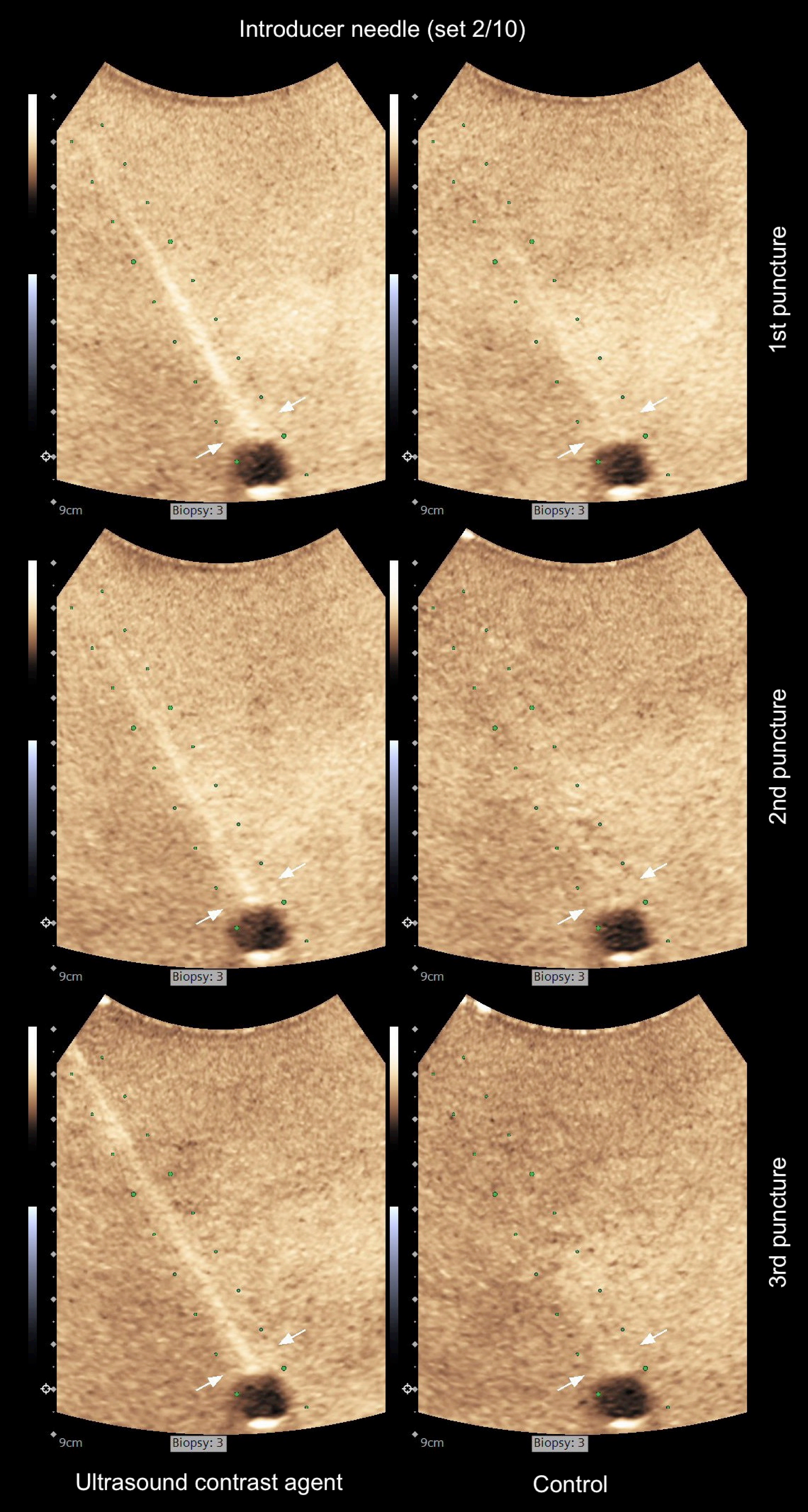
Introducer needle (set 2/10)
1st puncture
2nd puncture
3rd puncture
9cm
Biopsy: 3
Ultrasound contrast agent
Control

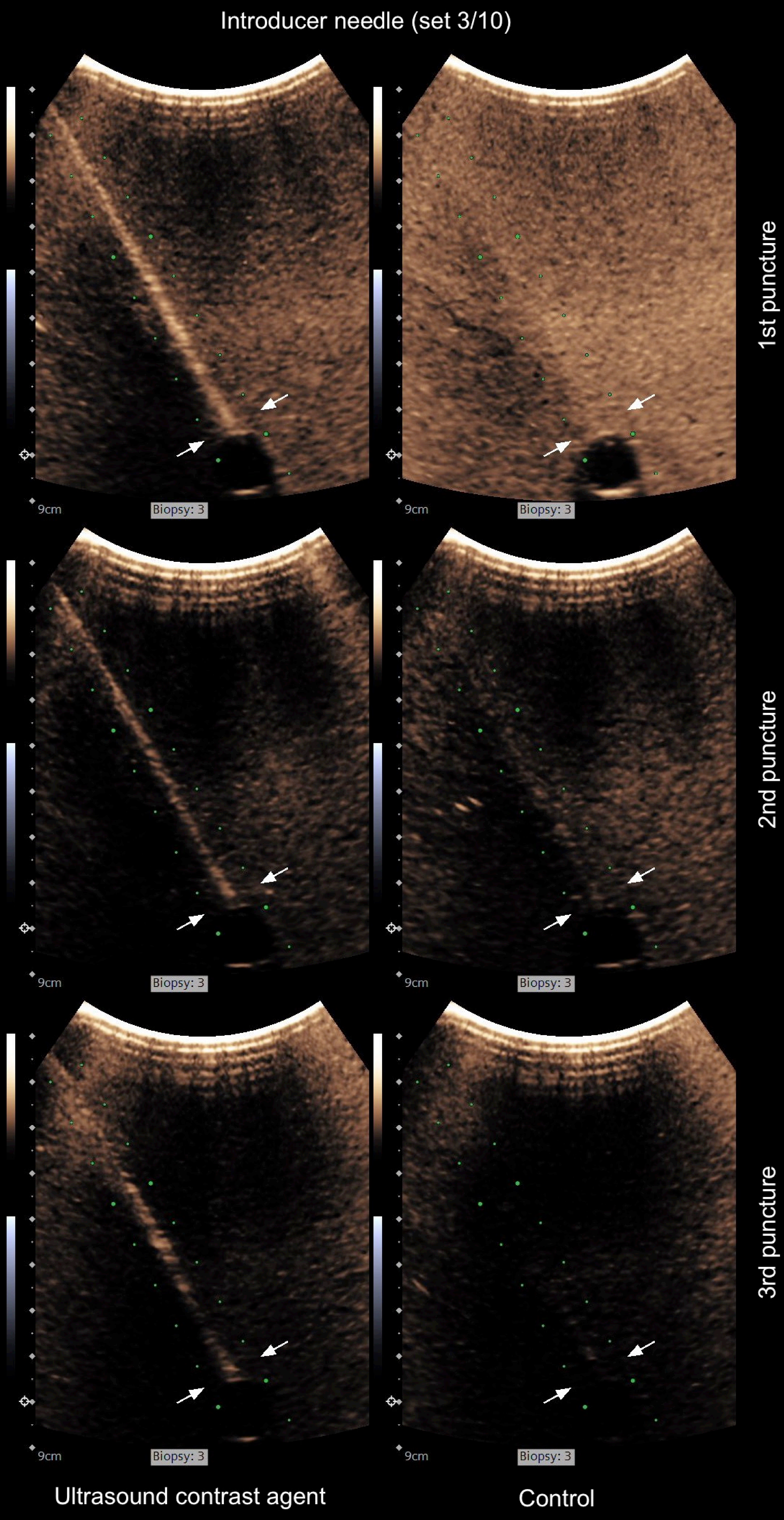
Introducer needle (set 3/10)
1st puncture
2nd puncture
3rd puncture
9cm
Biopsy: 3
Ultrasound contrast agent
Control

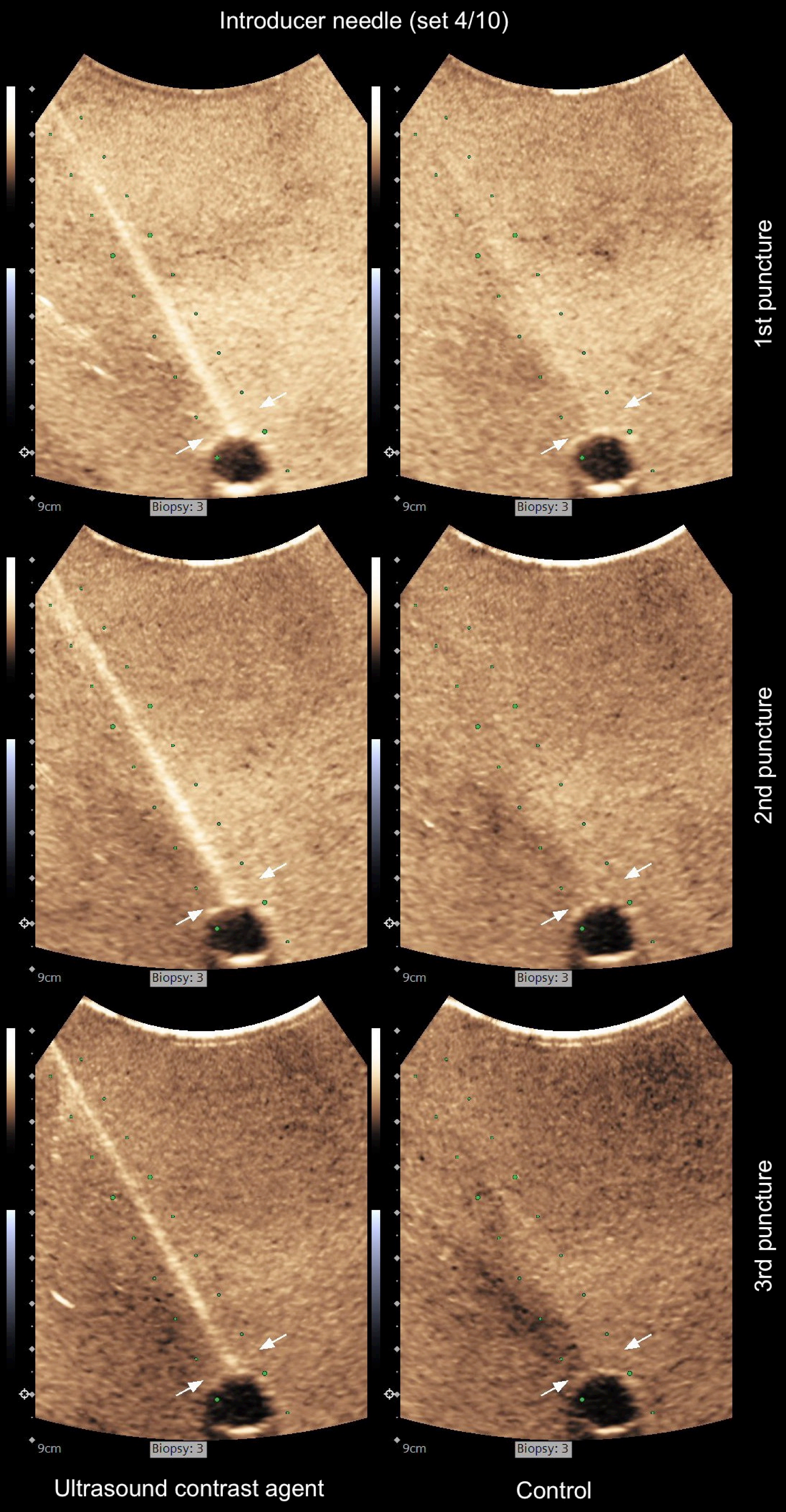

Introducer needle (set 4/10)
1st puncture
2nd puncture
3rd puncture
9cm
Biopsy: 3
Ultrasound contrast agent
Control

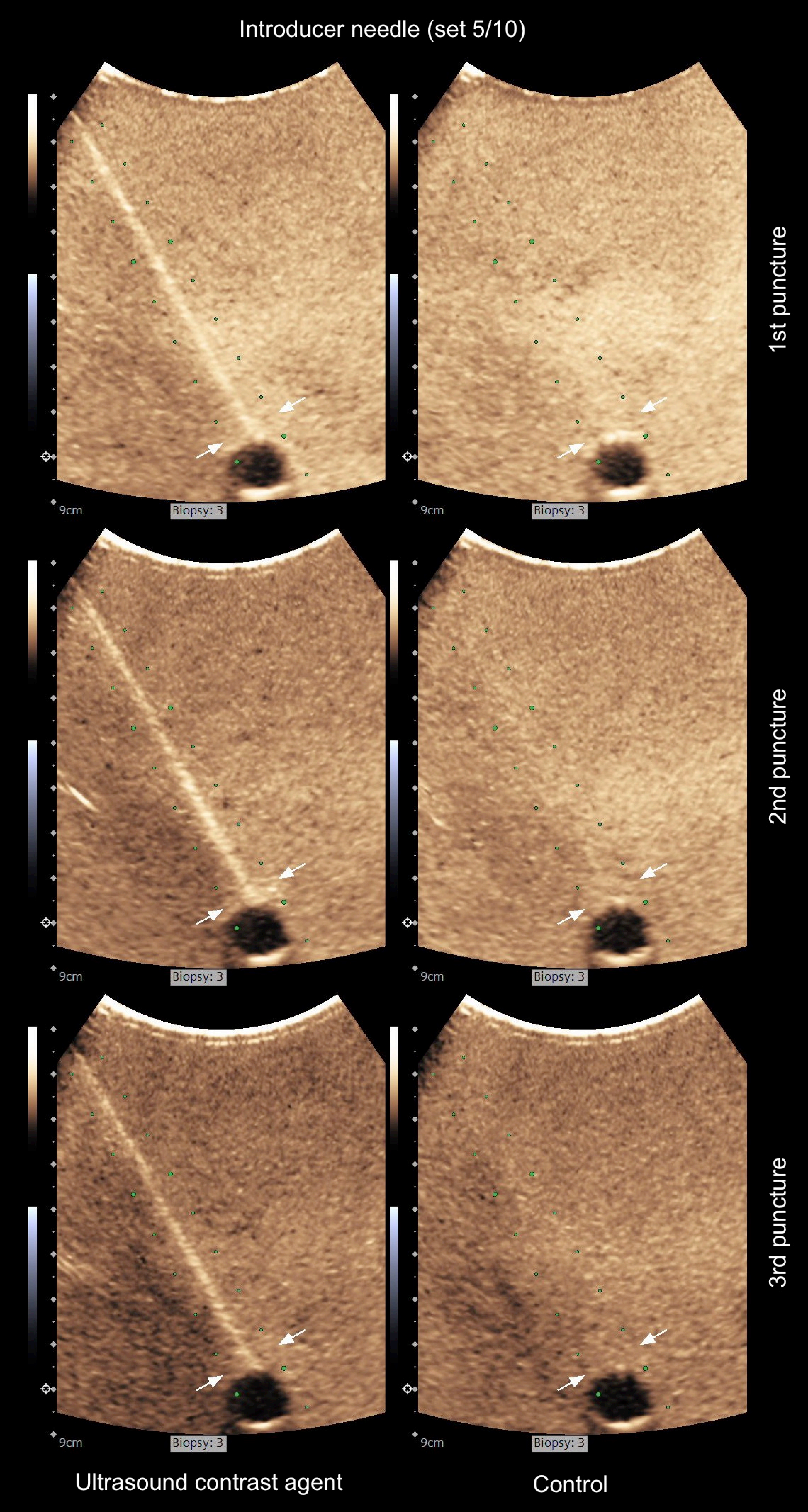

Introducer needle (set 5/10)
1st puncture
2nd puncture
3rd puncture
9cm
Biopsy: 3
Ultrasound contrast agent
Control

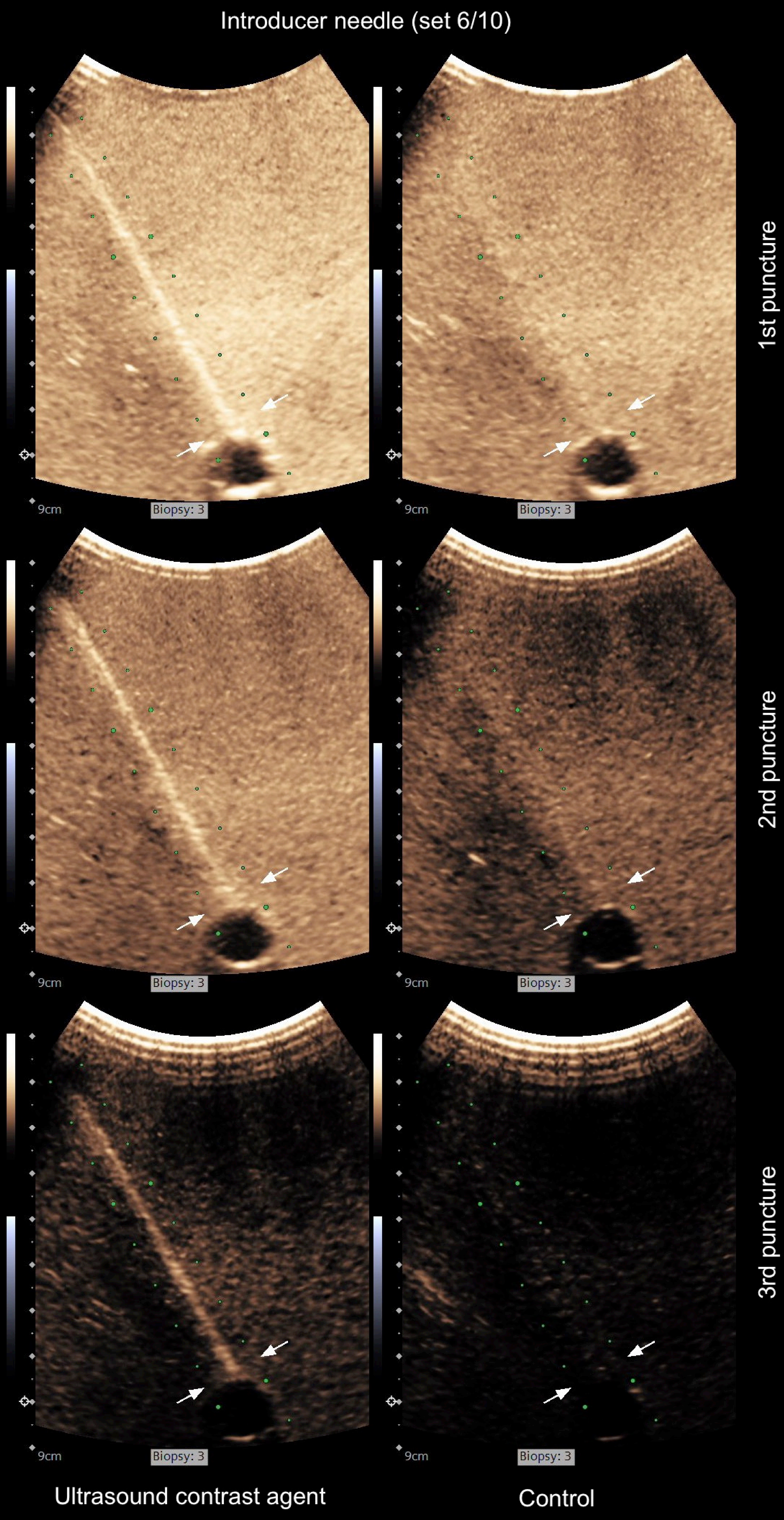
Introducer needle (set 6/10)
1st puncture
2nd puncture
3rd puncture
9cm
Biopsy: 3
Ultrasound contrast agent
Control

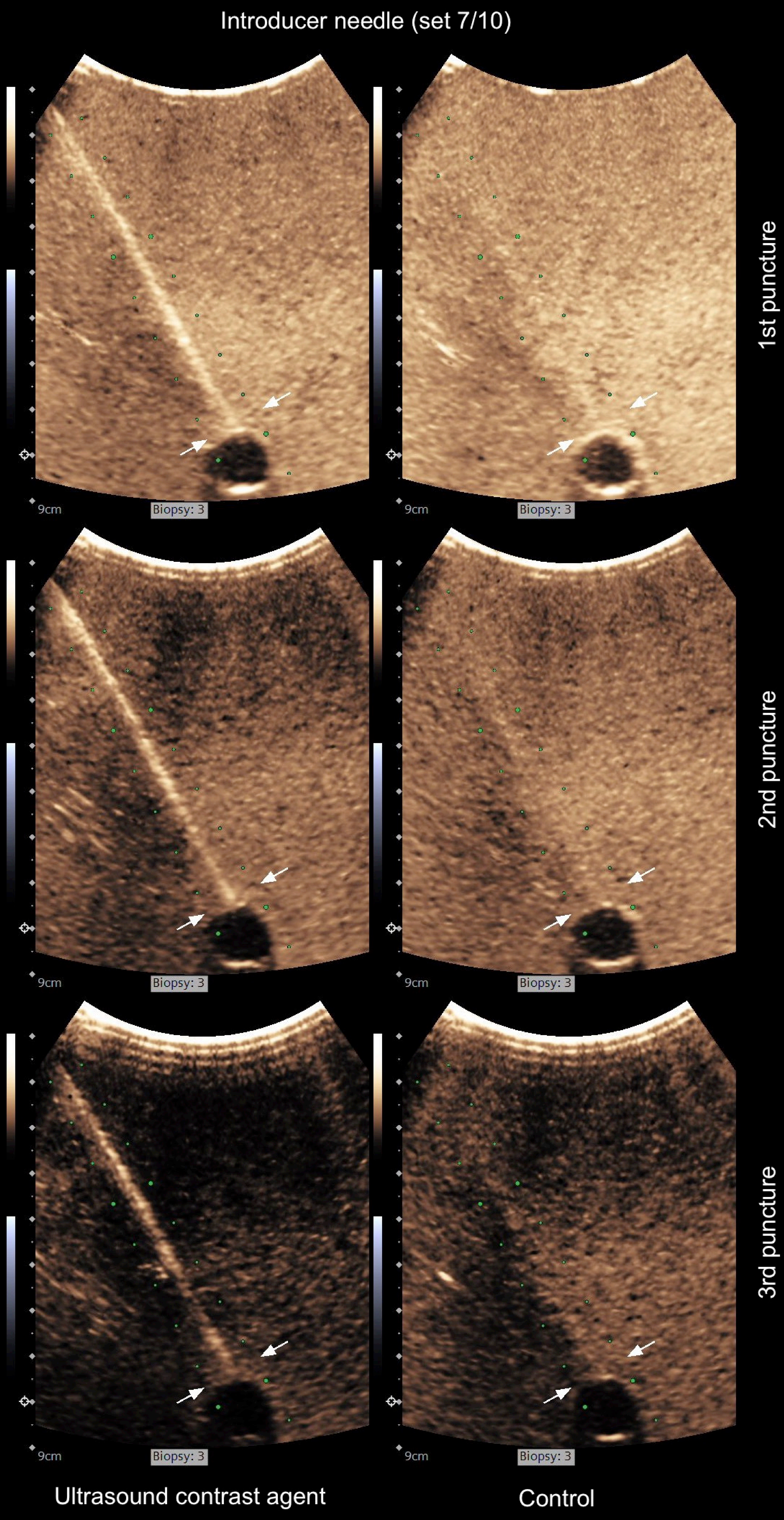
Introducer needle (set 7/10)
1st puncture
2nd puncture
3rd puncture
9cm
Biopsy: 3
Ultrasound contrast agent
Control

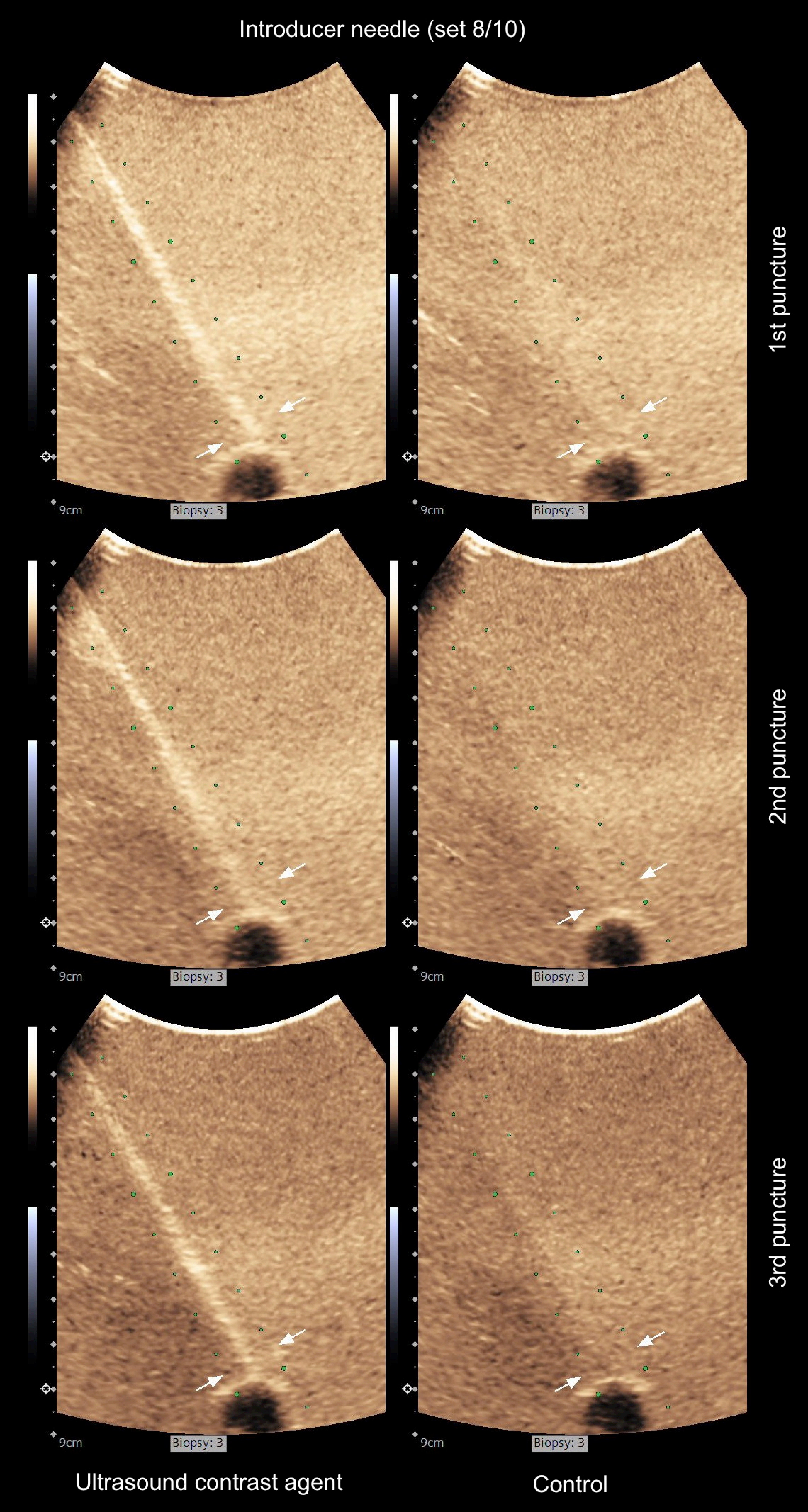
Introducer needle (set 8/10)
1st puncture
2nd puncture
3rd puncture
9cm
Biopsy: 3
Ultrasound contrast agent
Control

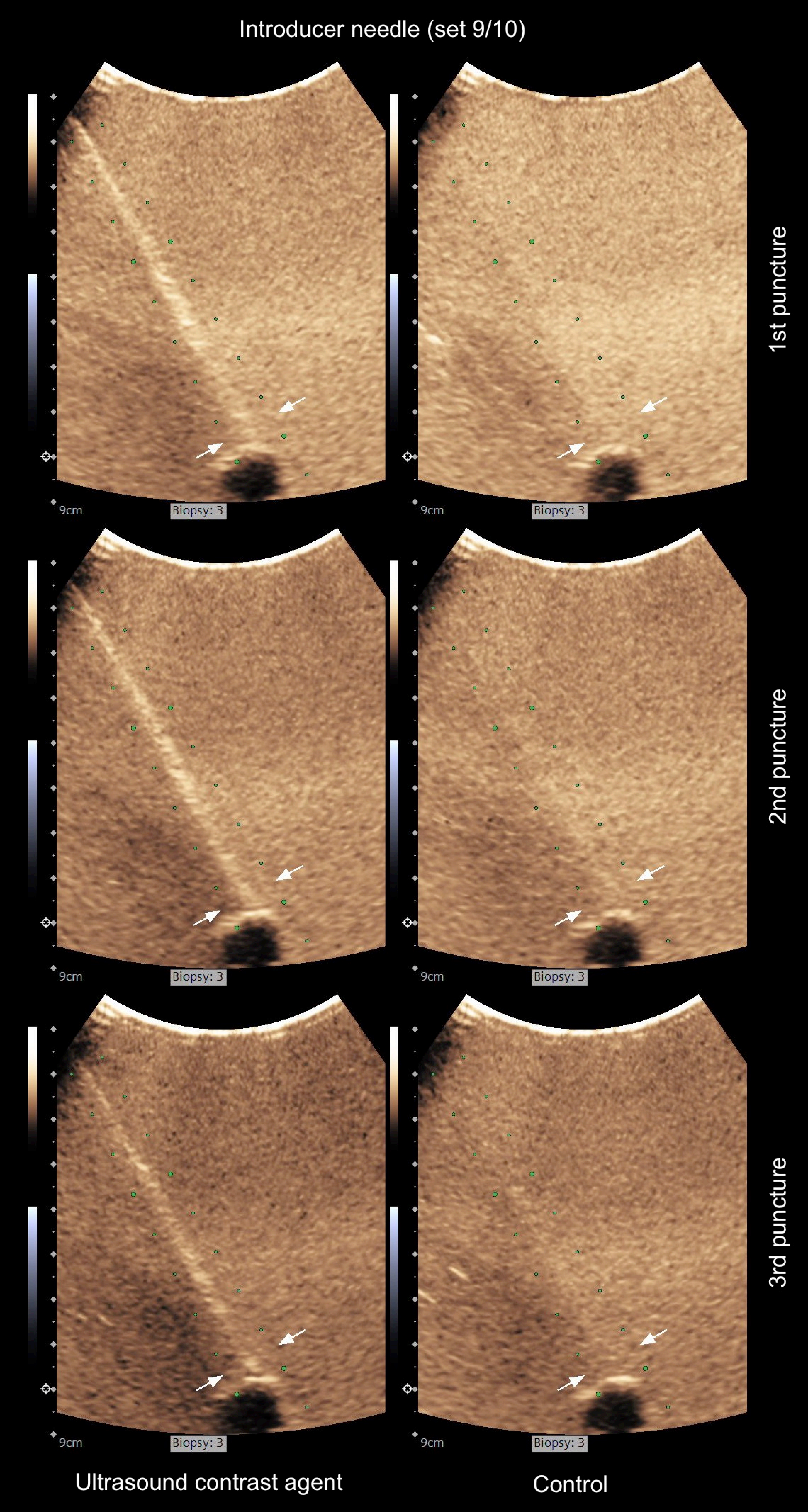

Introducer needle (set 9/10)
1st puncture
2nd puncture
3rd puncture
9cm
Biopsy: 3
Ultrasound contrast agent
Control

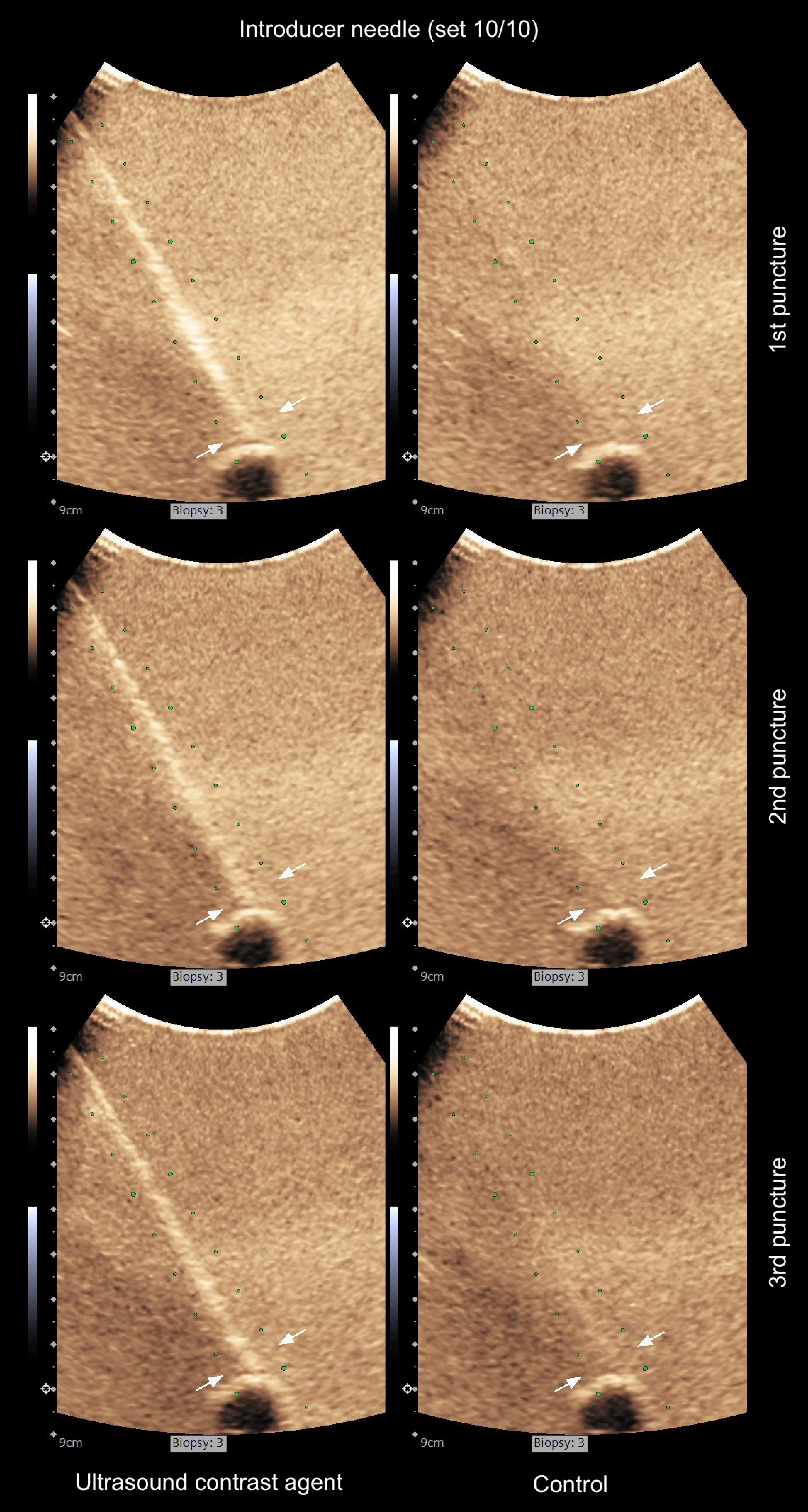

Introducer needle (set 10/10)
1st puncture
2nd puncture
3rd puncture
9cm
Biopsy: 3
Ultrasound contrast agent
Control
